# Mechanism of synergistic actin filament pointed end depolymerization by cyclase-associated protein and cofilin

**DOI:** 10.1038/s41467-019-13213-2

**Published:** 2019-11-22

**Authors:** Tommi Kotila, Hugo Wioland, Giray Enkavi, Konstantin Kogan, Ilpo Vattulainen, Antoine Jégou, Guillaume Romet-Lemonne, Pekka Lappalainen

**Affiliations:** 1 HiLIFE Institute of Biotechnology, University of Helsinki, FI-00014 Helsinki, Finland; 2 Université de Paris, CNRS, Institut Jacques Monod, 75013 Paris, France; 3 Department of Physics, University of Helsinki, FI-00014 Helsinki, Finland; 4 Computational Physics Laboratory, Tampere University, FI-33101 Tampere, Finland

**Keywords:** Cytoskeletal proteins, X-ray crystallography

## Abstract

The ability of cells to generate forces through actin filament turnover was an early adaptation in evolution. While much is known about how actin filaments grow, mechanisms of their disassembly are incompletely understood. The best-characterized actin disassembly factors are the cofilin family proteins, which increase cytoskeletal dynamics by severing actin filaments. However, the mechanism by which severed actin filaments are recycled back to monomeric form has remained enigmatic. We report that cyclase-associated-protein (CAP) works in synergy with cofilin to accelerate actin filament depolymerization by nearly 100-fold. Structural work uncovers the molecular mechanism by which CAP interacts with actin filament pointed end to destabilize the interface between terminal actin subunits, and subsequently recycles the newly-depolymerized actin monomer for the next round of filament assembly. These findings establish CAP as a molecular machine promoting rapid actin filament depolymerization and monomer recycling, and explain why CAP is critical for actin-dependent processes in all eukaryotes.

## Abstract (web summary)

The cofilin family proteins are actin disassembly factors but the disassembly mechanism is poorly understood. Here authors show that cyclase-associated-protein (CAP) works in synergy with cofilin to accelerate actin filament depolymerization by nearly 100-fold and reveal how CAP destabilizes the interface between terminal actin subunits.

## Introduction

The most prominent force-producing machinery in eukaryotic cells is the actin cytoskeleton. It powers diverse cellular processes, including migration, morphogenesis, and endocytosis through rapid polymerization of actin filaments at their barbed ends against the cellular membranes. In cells, the rapid assembly of actin filaments must be balanced by filament disassembly at their pointed ends to maintain the cytoplasmic pool of assembly-competent actin monomers. While the mechanisms of actin filament nucleation, polymerization, and capping^1–6^ are relatively well-established, we know significantly less about how actin filaments are disassembled to maintain actin filament growth^7^.

Several proteins, including the members of actin-depolymerizing factor (ADF)/cofilin and gelsolin families, are involved in the disassembly of actin filaments. Gelsolin family proteins can associate with both monomeric and filamentous actin in a Ca^2+^-dependent manner, and regulate the architecture of the actin cytoskeleton by severing and capping actin filaments^8–10^. ADF/cofilins, which are critical for actin dynamics in all eukaryotes tested so far, interact with both monomeric and filamentous actin, and accelerate cytoskeletal dynamics by severing actin filaments at the interface of bare and ADF/cofilin-decorated filament segments^11–14^. ADF/cofilins are present in all eukaryotes, and are critical for the maintenance of rapid actin filament turnover in cells^15^. Thus, ADF/cofilins multiply the number of filament ends that undergo depolymerization. ADF/cofilins also modestly accelerate actin filament depolymerization^16–18^. However, whether ADF/cofilin-catalyzed actin filament severing and slow depolymerization are sufficient for rapid actin dynamics in cells, or if additional dedicated actin filament depolymerization factors are needed, has remained an outstanding question. Moreover, the mechanism by which the newly depolymerized actin monomers are recycled from ADF/cofilins for the next round of filament assembly is incompletely understood.

Another actin-binding protein implicated in filament disassembly is cyclase-associated protein (CAP). It is conserved in evolution from protozoan parasites through yeasts and plants to animals, and is thus among the small number (<10) of core actin-binding proteins present in all eukaryotes^19–21^. Genetic studies demonstrated that CAP is critical for actin-dependent cellular and developmental processes in all organisms tested so far, and its depletion results in abnormal accumulation of actin filaments and diminished actin filament turnover rates^22–29^. Moreover, altered expression levels of CAP are linked to various cancers^30,31^.

CAP is a relatively large multidomain protein, which can self-assemble into oligomers, most likely hexamers^32,33^. CAP can re-charge ADP-actin monomers with ATP through its C-terminal half that harbors Wiscott Aldrich Syndrome protein homology 2 (WH2) and CAP-retinitis pigmentosa (CARP) domains^34^. Moreover, the N-terminal half of CAP (N-CAP), consisting of oligomerization (OD) and helical folded (HFD) domains^35^, binds cofilin/actin monomer complexes^36^, and can enhance disassembly of actin filaments together with ADF-H domain proteins; twinfilin and ADF/cofilin^32,37–39^ (Supplementary Fig. 1a). However, whether CAP promotes actin filament disassembly by accelerating filament severing or by promoting filament depolymerization has remained enigmatic. Moreover, the mechanism by which the different functions of CAP, actin filament disassembly and monomer re-charging, are coordinated is incompletely understood. Thus, the molecular principles by which this ubiquitous and essential actin-regulatory protein promotes cytoskeletal dynamics have remained elusive.

Here, we reveal that CAP promotes rapid actin filament disassembly by accelerating pointed end depolymerization of cofilin-decorated actin filaments. By determining the crystal structure of CAP’s HFD domain in complex with actin and an ADF-H domain, combined with atomistic molecular dynamics simulations, we uncover the molecular mechanism by which CAP depolymerizes actin filaments and recycles actin monomers for the next round of filament assembly.

## Results

### CAP interacts with the pointed end of actin

**Fig. 1:**
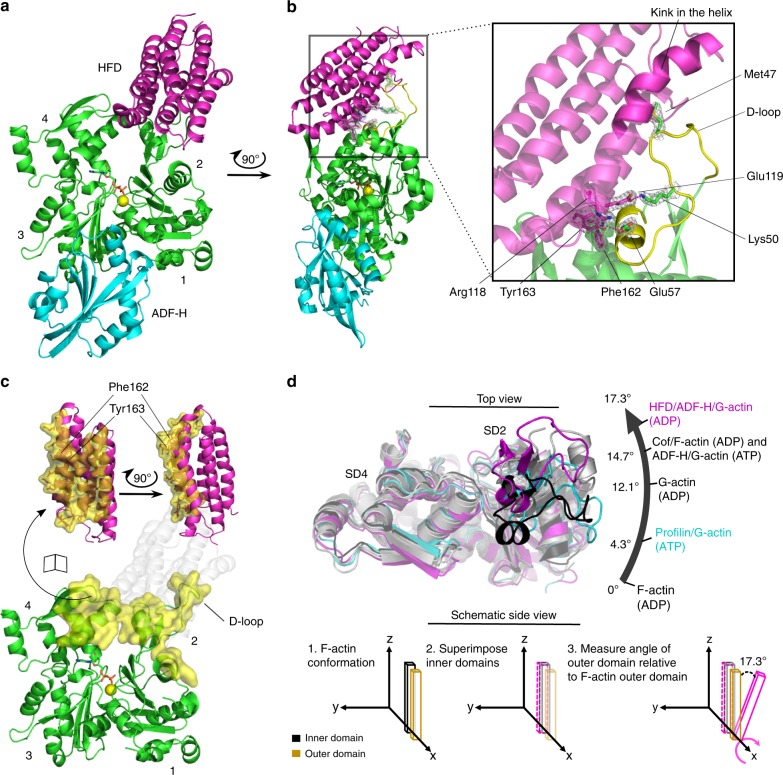
The crystal structure of the HFD domain of CAP in complex with ADP-actin and ADF-H domain. **a** The HFD domain of CAP (magenta) binds to the pointed end of an ADP-actin monomer (green) between subdomains 2 and 4, whereas twinfilin’s ADF-H domain (cyan) binds to the barbed end of the monomer between subdomains 1 and 3. **b** Highlighted in yellow are actin’s D-loop and the adjacent α-helix (residues 55–61), which are spatially coordinated by Tyr162 and Phe163 of the HFD domain. Lys50 and Glu57 of actin form salt bridges with Glu119 and Arg118 of the HFD domain, respectively. Met47 of actin fits into a hydrophobic cavity formed by a kink in the contacting α-helix of the HFD domain. Selected residues are presented in their corresponding 2*F*_0_ – *F*_C_ (*σ* = 1.0) electron density map. **c** The HFD domain covers an extensive binding surface of 1208 Å^2^ (yellow) predominantly on actin subdomain 2. **d** Different conformational states of actin monomers analyzed by comparing the twist between actin structures superimposed on their inner domains (subdomains 3 and 4) and measuring the angle of the outer domain (subdomain 1) relative to the ADP-state of F-actin (PDB = 6djo). The HFD domain-bound actin monomer displays a larger rotation of the outer domain compared to the other indicated structures (PDB = 2btf for profilin/G-actin; 1j6z for G-actin; 3daw for ADF-H domain/G-actin; 5yu8 actin monomer from a cofilin-decorated actin filament). See Supplementary Fig. 1e-f.

The N-terminal HFD domain of CAP is critical for actin filament disassembly in vitro and in cells^32^. However, the structure of this domain does not resemble other known actin-binding protein domains^35^, and thus the mechanism by which it interacts with actin has remained unknown. To reveal the structural mechanism by which CAP binds actin to regulate cytoskeletal dynamics, we crystallized the HFD domain of mouse CAP1 alone (Table 1, Supplementary Fig. 1b, c), as well as in complex with ADP-G-actin and ADF-H domain from twinfilin (Fig. 1a; Table 1). The ADF-H domain of twinfilin is structurally similar to ADF/cofilins, and interacts with actin through the same interface^40,41^, but allows crystal formation due to its monomer sequestering function. The 1.95 Å resolution crystal structure of the tripartite complex revealed that the HFD domain binds to the pointed end of an actin monomer, between subdomains 2 (SD2) and 4 (SD4) (Fig. 1a). In comparison to other proteins associating with the pointed end of actin, including tropomodulin, which prevents elongation of actin filaments and β-thymosin, which sequesters actin monomers^42–44^, HFD domain binding heavily relies on contacts with SD2 of actin (Fig. 1b, c; Supplementary Fig. 1d). Intriguingly, within this interface we observed a tailor-made hydrophobic cavity in the contacting α-helix of the HFD domain that coordinates Met47 of actin (Fig. 1b, c) and thus locks the D-loop to an upright orientation. Moreover, interaction of the HFD domain with actin is spatially coordinated by specific aromatic residues and ion pairs formed between the HFD domain and the α-helix of actin (residues 55–61) adjacent to the D-loop (Fig. 1b). Accordingly, mutating Phe162 and Tyr163 in the HFD domain disrupted the interaction with twinfilin/actin monomer and cofilin/actin monomer complexes, demonstrating that these interactions are indeed critical for binding of the HFD domain to actin (Supplementary Fig. 2a−c).

**Table 1:** Crystallographic data collection and refinement statistics.

	HFD domain	HFD domain bound to ADF-H/ADP-actin
Data collection		
Space group	P 21 21 21	P 1 21 1
Cell dimensions		
*a*, *b*, *c* (Å)	82.51, 91.41, 106.87	87.37, 54.49, 87.83
*α*, *β*, *γ* (°)	90, 90, 90	90, 93.61, 90
Resolution (Å)	41.26−2.37 (2.46−2.37)^a^	41.45−1.95 (2.02−1.95)^a^
* R*_merge_	0.138 (1.786)	0.088 (1.073)
* I*/*σI*	10.6 (1.31)	12.9 (1.58)
Completeness (%)	99.8 (99.76)	99.9 (99.87)
Redundancy	7.0 (7.3)	6.4 (6.2)
Refinement		
Resolution (Å)	41.26−2.37	41.45−1.95
No. reflections	33,463	60,448
* R*_work_/*R*_free_	0.186/0.23	0.166/0.194
No. atoms		
Protein	5565	5417
Ligand/ion	18	70
Water	115	652
* B*-factors		
Protein	65.1	44.2
Ligand/ion	73.7	51.9
Water	55.9	51.4
R.m.s. deviations		
Bond lengths (Å)	0.014	0.015
Bond angles (°)	1.71	1.67
Ramachandran (%)		
Favored	98.9	98.2
Outliers	0	0.15
PDB code	6RSQ	6RSW

^a^Values in parentheses are for highest-resolution shell

Interestingly, the HFD domain interacts with the cofilin/actin complex with higher affinity compared to bare actin^36^ (Supplementary Fig. 2c), although it does not make a direct contact with the ADF-H domain. Analysis of various actin structures revealed that actin is more twisted in the ADF-H domain/G-actin and in ADF-H domain/G-actin/HFD domain complexes compared to structure ADP-G-actin (Fig. 1d; Supplementary Fig. 1e, f). This provides a plausible explanation for the binding-preference of HFD domain for cofilin/G-actin. Together, these structural data reveal that the HFD domain of CAP binds to the pointed end of actin through a unique mechanism.

### CAP promotes actin filament pointed end depolymerization

**Fig. 2:**
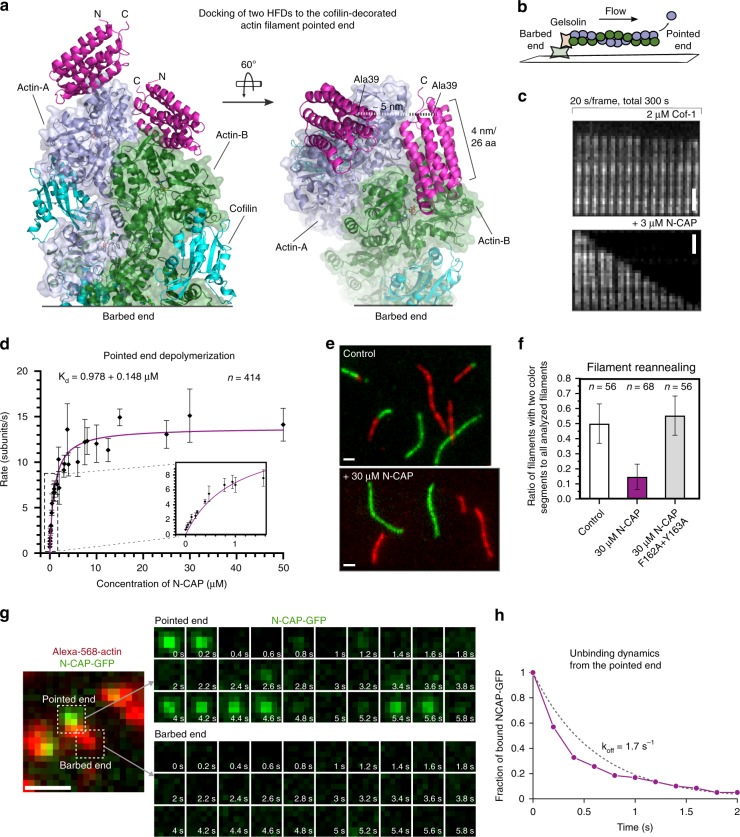
CAP catalyzes pointed end depolymerization of cofilin-decorated actin filaments. **a** The actin monomers in a cofilin-decorated filament (PDB = 5yu8) display a similar conformation to the one in our crystal structure (Fig. 1d), allowing docking of two HFD domains of CAP to the two pointed end actin subunits without steric clashes. Rotated view shows that the N-termini of the two HFD domains (Ala39) are within 5 nm, suggesting the filament-bound CAP can still oligomerize. **b** A schematic of the actin filament pointed end depolymerization assay. **c** Representative kymographs showing the pointed end depolymerization of cofilin-decorated filaments in the absence (top) and presence (bottom) of 3 μM N-CAP. **d** The pointed end depolymerization activity of N-CAP fitted by first-order saturation suggests a *K*_d_ of 0.978 + 0.148 μM for N-CAP binding to the pointed end of cofilin-decorated actin filament. Error bars, S.D., *n* ≥ 5 for each point. **e** Examples of actin filament reannealing experiments, where actin filaments labeled with different colors were mixed and imaged. **f** Quantification of the reannealing experiments performed in the absence of N-CAP (control) and presence of wild-type N-CAP or a mutant defective in binding to the actin monomer pointed end (see Supplementary Fig. 2a−c). Reactions contained 8 μM actin and 30 μM N-CAP. Bars depict the ratio of two-colored filaments to all analyzed filaments. Error bars, 95% CI in binominal distribution. **g** Association of N-CAP-GFP with filament pointed ends. A solution of 200 nM F-actin (10% Alexa-568, 1% biotin), 100 nM N-CAP-GFP, 2 µM cofilin-1, 4 nM capping protein, and 0.2% methylcellulose, was injected into an open chamber. Left: A typical actin filament (red). The pointed end was designated as the one with frequent N-CAP-GFP binding (green), because the filaments were capped at barbed ends. Right: Stream acquisition of GFP signal, imaged with 5 frames/second. The binding of N-CAP-GFP was detected in ~17% of the total number of frames for filament pointed ends (*n* = 20 filaments, 60 frames). **h** Unbinding dynamics of N-CAP-GFP from the pointed end (*n* = 20 filaments, 73 binding events). Scale bars, 2 μm. Source data are provided as a Source Data file.

Next, we examined how CAP could accelerate the disassembly of actin filaments. Because the HFD domain of CAP binds to the pointed end of an actin monomer, and the conformation of actin in our tripartite complex is very similar to the one of cofilin/F-actin (Fig. 1d), we examined if CAP could associate also with the pointed end of a cofilin-decorated actin filament. Two HFD domains could indeed be docked to the pointed end of the cofilin/F-actin structure^41^ in a way that their actin interaction surfaces are preserved from our crystal structure (Fig. 2a). However, the HFD domain could not be docked to the side of either bare^45^ or cofilin-decorated actin filament (Supplementary Fig. 3a, b). This suggests that CAP might not sever actin filaments as previously reported^32,37^, but may instead enhance their disassembly through interaction with the filament pointed end.

To test the hypothesis that CAP might enhance actin filament disassembly by associating with filament pointed ends, we performed actin filament pointed end depolymerization assays by using a single-filament microfluidics approach^46^ (Fig. 2b). As reported earlier^16,18^, cofilin-saturated actin filaments underwent slightly more rapid depolymerization from their pointed ends compared to bare actin filaments under physiological conditions. Strikingly, addition of N-CAP dramatically accelerated pointed end disassembly of cofilin-decorated actin filaments (Fig. 2c). At saturating conditions, the pointed end depolymerization of cofilin-decorated ADP-actin filaments was accelerated by N-CAP up to ~30-fold, and compared to bare ADP-actin filaments the depolymerization rate was increased by nearly 100-fold in the presence of both cofilin and N-CAP (Fig. 2d). By determining the depolymerization rates at different N-CAP concentrations, we estimated a *K*_d_ of ~1 μM for the interaction of N-CAP with cofilin-saturated filaments.

We further analyzed the ability of N-CAP to bind to actin filament pointed ends by performing actin filament reannealing experiments. The presence of N-CAP leads to a clear inhibition of filament reannealing, whereas mutations in the actin-binding interface of the protein diminished this effect (Fig. 2e, f). Importantly, by using biotin-labeled actin filaments that were anchored to the chamber surface, and a GFP-tagged version of N-CAP, we could directly detect the association of N-CAP at filament pointed end with a residence time of ~0.4 s (Fig. 2g, h).

**Fig. 3:**
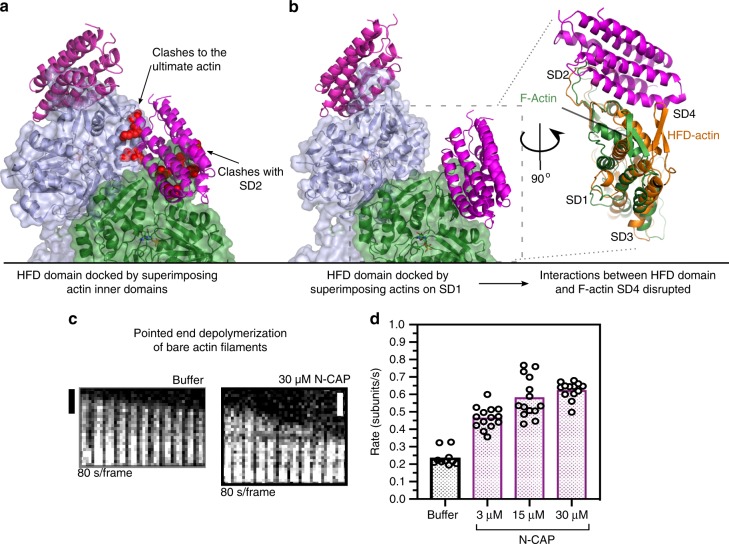
Conformation of the bare filament pointed end is not favorable for binding of CAP. **a** Superimposition of ADP-actin from the HFD domain/ADF-H domain/actin structure to the pointed end of a bare ADP-actin filament (PDB = 6djo) by aligning subdomains 3 and 4 of actins causes clashes between the D-loop of actin and the HFD domain. As a result of nonoptimal docking, the HFD domain bound to the penultimate actin monomer collides with the ultimate actin monomer in the filament. Steric clashes are indicated with red spheres. **b** Superimposition of ADP-actin from the HFD domain/ADF-H domain/actin structure to the pointed end of a bare actin filament by aligning subdomain 1 of actins does not cause clashes between actin and the HFD domain. However, the conformation of bare filamentous actin (green) is not optimal for HFD domain binding when compared to the crystallized actin conformation (orange). This is due to the difference in the twist of the actin (see Fig. 1d) that decreases contacts between the HFD domain and actin subdomain 4. **c** Examples of bare actin filament pointed end depolymerization assays (by single-filament microfluidics approach) performed in the absence and presence of 30 μM N-CAP. **d** Quantification of the rate of pointed end depolymerization of bare actin filaments at different N-CAP concentrations. Circles represent the pointed end depolymerization rates of individual actin filaments. Scale bars 1 μm. Source data are provided as a Source Data file.

We also tested whether N-CAP could enhance the pointed end depolymerization of bare actin filaments. However, docking of the HFD domain–actin complex to the pointed end of the bare actin filament leads to nonoptimal contacts or clashes with actin, suggesting less favored interaction of the HFD domain with the pointed end of the bare actin filament compared to the cofilin-decorated filament (Fig. 3a, b). It is important to note that the atomic structures of actin filament ends have not been determined, and thus we assumed that the pointed ends of actin filaments adopt similar conformations as observed in the monomers at central regions of actin filament. Consistent with these structural analyses, N-CAP enhanced the pointed end depolymerization of bare actin filaments much less efficiently compared to cofilin-decorated filaments (Fig. 3c, d). Similarly, we observed the cofilin-dependency for N-CAP-mediated actin filament disassembly also in a bulk actin disassembly assay (Supplementary Fig. 2d).

Earlier work suggested that the N-terminal half of CAP can accelerate the severing of partially cofilin-decorated actin filaments^32,33,37^. Thus, we examined the effects of mouse N-CAP on actin filament severing. Consistent with the inability of HFD domain to be docked to the side of an actin filament, we could not detect any increase in severing activity induced by N-CAP on partially decorated cofilin-actin filaments using single-filament microscopy, and only very modest increase in filament severing was observed with full-length CAP (Supplementary Fig. 3c, d). Thus, our biochemical and structural data show that CAP does not promote robust actin filament severing, but drives rapid actin filament disassembly by dramatically accelerating filament pointed end depolymerization.

### Mechanism of CAP-catalyzed actin filament depolymerization

**Fig. 4:**
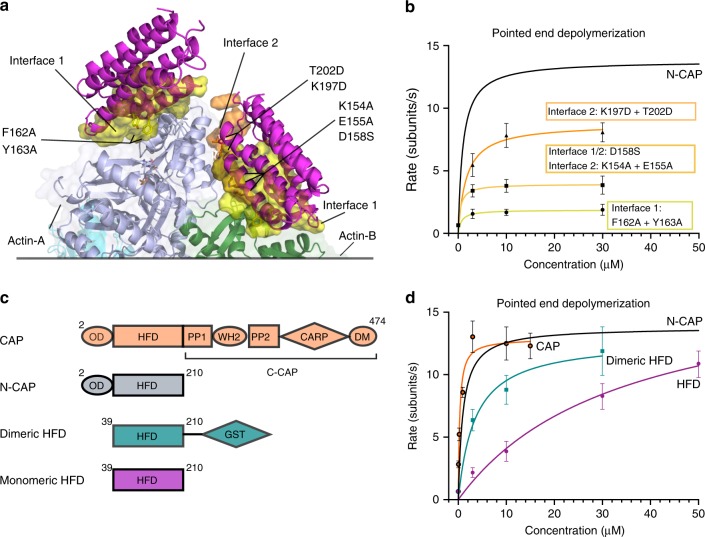
Molecular mechanism of actin filament pointed end depolymerization by CAP. **a** Energy-minimized model of two HFD domains bound to a cofilin-decorated actin filament pointed end. HFD domains bind actin through two interfaces: The one determined in our crystal structure (interface 1, yellow), and interface 2 (orange), which forms between the ultimate actin (actin A) and the HFD domain bound to the penultimate actin (actin B). Residues shown in sticks were mutated to validate the binding interfaces. **b** Cofilin-decorated actin pointed end depolymerization rates facilitated by N-CAP mutants as determined by single filament microfluidics. *n* ≥ 5 for each concentration. **c** Different CAP constructs used in this study. The dimeric HFD domain lacks the oligomerization domain (OD), but was fused to glutathione-*S*-transferase (GST) from its C-terminus to form dimers (see Supplementary Fig. 4). PP=polyproline region, DM=dimerization motif. **d** Pointed end depolymerization rates of cofilin-decorated actin filaments facilitated by different CAP constructs. The isolated HFD domain is inefficient in depolymerizing cofilin-decorated actin filaments at low concentrations, whereas dimerization of this domain through GST enhances its pointed end depolymerization activity. Oligomeric full-length CAP and N-CAP efficiently accelerate actin filament depolymerization already at low protein concentrations. *n* ≥ 5 for each CAP concentration. Error bars present S.D. Source data are provided as a Source Data file.

To elucidate the mechanism by which CAP drives actin filament pointed end depolymerization, we performed structure-guided mutagenesis on N-CAP. Both HFD domains, docked to the pointed end of a cofilin-decorated actin filament, associate with subdomains 2 and 4 of the terminal actins via the surface identified by our crystal structure (Fig. 4a, interface 1). The HFD domain docked to the penultimate actin molecule (Actin B) also associates with the ultimate actin molecule (Actin A) through a second surface (interface 2) that does not correspond to the binding-surface identified in the crystal structure (Fig. 4a). Mutations in surface-exposed residues at both interfaces resulted in defects in N-CAP’s ability to depolymerize actin filaments at their pointed ends, providing evidence that CAP indeed uses these two interfaces for its high affinity association with filament pointed ends (Fig. 4b; Supplementary Fig. 4a, b).

We next examined the roles of CAP’s different domains in actin filament depolymerization (Fig. 4c). Both the oligomeric full-length CAP and N-CAP were equally efficient in accelerating pointed end depolymerization of cofilin-saturated filaments, whereas in the case of monomeric HFD domain >20-fold higher concentrations were needed for strong acceleration of filament depolymerization (Fig. 4d; Supplementary Fig. 4c−e). To elucidate whether the difference in depolymerization activities between the isolated HFD domain and N-CAP, which also contains a short OD domain, is due to oligomerization or possible other function of the N-terminal OD domain, we generated an artificial HFD domain dimer by fusing glutathione-*S*-transferase to its C-terminus (Fig. 4c). Interestingly, the dimeric HFD domain was able to strongly accelerate actin filament depolymerization at much lower concentration when compared to the monomeric HFD domain. These data demonstrate that the HFD domain is the functionally minimal unit for actin filament pointed end depolymerization, and that its oligomerization decreases the effective protein concentration needed for this activity. Thus, we propose that filament pointed end depolymerization requires simultaneous association of two HFD domains with the two terminal actins (Fig. 4d). Together, these data show that the HFD domain is the main functional unit of CAP for actin filament depolymerization, and that dimerization/oligomerization of the HFD domains increases the efficiency of depolymerization, possibly through increased ability to bind to the pointed end of actin filaments through the effect of avidity.

### CAP destabilizes the pointed end of actin filament

**Fig. 5:**
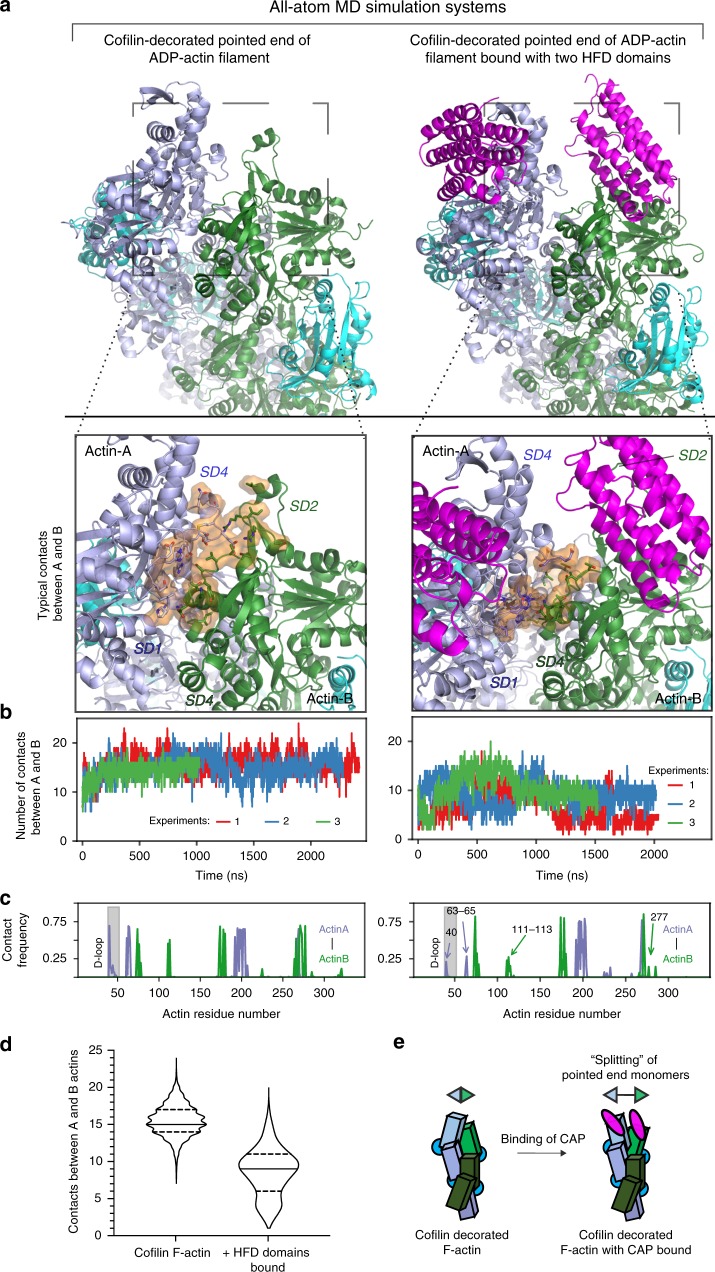
CAP destabilizes the interface between the terminal actins at the filament pointed end. **a** All-atom molecular dynamics simulations of cofilin-decorated actin filaments (consisting of four actins and three cofilins; see Supplementary Fig. 5a) in the absence of CAP, and in the presence of two HFD domains bound to filament pointed ends. MD simulations show that the interface between terminal actins of the filament opens in the presence of HFD domains, as indicated by the decrease in the number of contacts between these subunits. **b** The number of contacts between actins A and B at the pointed end from three simulations (started from three independently generated configurations; see Methods) shown as a function of time. Left: Cofilin-decorated ADP-actin pointed end. Right: With two HFD domains bound to the cofilin-decorated ADP-actin pointed end. The number of contacts was defined as the number of residues from both chains within 3 Å of each other. **c** The contact frequencies of individual residues between actin A and actin B are shown. Left: The cofilin-decorated ADP-actin filament pointed end. Right: With two HFD domains bound to cofilin-decorated ADP-actin filament pointed end. The frequency of contacts was averaged over three simulations. The residues that underwent a major reduction in the contact frequency in the presence of the HFD domains are indicated by arrows. For detailed analysis of contacts between other actins and HFD domains, see Supplementary Fig. 5e−f. **d** Violin plot displaying the distribution of the number of contacts between A and B actins from three independent simulations of cofilin-decorated filament pointed ends in the presence and absence of HFD domains. The first 500 ns from simulations were considered as an equilibration period, and were not included in the plot. 75 and 25% quartiles and median value are shown. **e** A schematic presentation of the CAP-induced pointed end destabilization suggested by the biochemical data and atomistic simulations. Source data are provided as a Source Data file.

To understand the molecular principles by which CAP accelerates actin monomer dissociation from filament pointed ends, we applied atomistic molecular dynamics (MD) simulations. These were performed on a pointed end segment of cofilin-decorated actin filament, both in the presence and absence of HFD domains (three repeats with total lengths of ~5.6 and ~5.8 μs, respectively; Fig. 5a, Supplementary Fig. 5a; Supplementary Table 1). Simulations of the cofilin-actin filament demonstrated that the two HFD domains indeed associate stably with the terminal actins in the configuration captured in the crystal structure (Supplementary Fig. 5b). Remarkably, the binding of HFD domains drastically reduced the number of contacts between the terminal actins in all simulations (Fig. 5b–d; Supplementary Fig. 5c, d). The most pronounced reductions in the frequencies of contacts were between the subdomains 1 and 4 of actin A with the subdomains 4 and 1 of actin B, respectively (Fig. 5c; Supplementary Fig. 5e−f). This resulted in a more open and dynamic actin filament pointed end compared to the HFD-free filament (Supplementary Movie 1). On the other hand, the interfaces between other actin subunits were largely unchanged, suggesting that CAP only destabilizes the terminal actin subunit in the filament pointed end (Supplementary Fig. 5e). Removal of the HFD domains during the simulation rapidly restored the contacts between the terminal actins (Supplementary Fig. 5c). Moreover, simulations where the HFD domains were docked to cofilin-actin filaments without optimizing the D-loop conformation to match with the one determined in our crystal structure were ineffective in destabilizing the interface (Supplementary Fig. 5c), suggesting the conformation of actin observed in the crystal structure is required for this effect.

Overall, these simulations, together with our mutagenesis data, provide evidence that binding of two HFD domains to the pointed end of actin filament, in the mode identified in our crystal structure, destabilizes the filament pointed end and forces the two terminal actin subunits to separate from each other (Fig. 5e; Supplementary Fig. 5d). We propose that this leads to the dissociation of the terminal actin subunit from the filament pointed end, and hence promotes the depolymerization of a cofilin-decorated actin filament.

### Mechanism of CAP-catalyzed actin monomer recycling

**Fig. 6:**
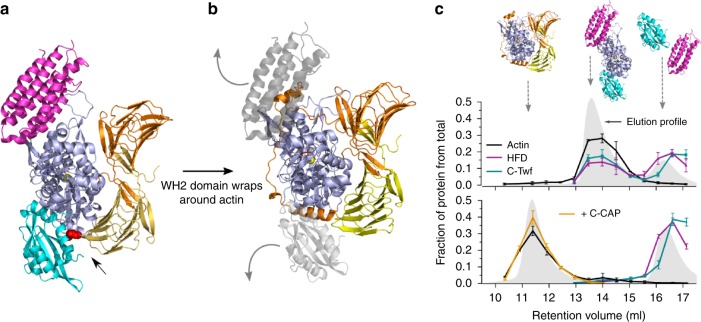
Mechanism of actin monomer recycling by CAP. **a** The C-terminal half of CAP is composed of CARP and WH2 domains and catalyzes ADP-to-ATP nucleotide exchange on actin monomers. The HFD domain (magenta) binds to an actin monomer (blue) using a different interface compared to the dimeric CARP domain of CAP (orange/yellow), which binds to the backside of actin monomers. A small steric clash between the CARP domain and ADF-H domain (cyan) is indicated with an arrow. **b** Based on prior structural data on WH2 domains^44^, mutagenesis^34^ and atomistic molecular dynamics simulations^34^, the WH2 domain of CAP binds to actin using an interface that overlaps with the ones of the HFD and ADF-H domains (light gray). **c** Gel filtration assay showing the formation of a tripartite complex between HFD domain, ADF-H domain, and ADP-G-actin (upper panel). Line profiles present the abundance of the proteins in each gel filtration fraction as analyzed by SDS-PAGE, and the gray area corresponds to the elution profile. Upon addition of the C-terminal half of CAP (C-CAP), the HFD and ADF-H domains are released from actin, and a complex between C-CAP and the actin monomer forms (lower panel). *n* = 3; error bars present S.D. For details, see Supplementary Fig. 6, and source data are provided as a Source Data file.

To elucidate whether the two main functions of CAP, actin filament depolymerization and monomer re-charging, could work together to enhance actin dynamics, we performed further structural analysis of different actin-bound states of CAP. We observed that the CARP domain of CAP, which binds ADP-actin monomers to promote nucleotide exchange^34^, interacts with actin using a different surface compared to the HFD domain (Fig. 6a). These data suggest that the newly depolymerized ADP-actin monomer could be first handed over from the HFD domain to the CARP domain. However, the adjacent WH2 domain occupies an interface on actin that clashes with the ones of the ADF-H and HFD domains (Fig. 6b), and this could lead to efficient replacement of the HFD domain and cofilin/twinfilin by the WH2 domain of CAP. To test this hypothesis, we performed gel filtration and native PAGE experiments, which revealed that the C-terminal half of CAP, and also the isolated CARP domain to a lesser extent, can indeed compete the actin monomer from the twinfilin/HFD domain and cofilin/HFD domain complexes (Fig. 6c; Supplementary Fig. 6). This leads to the release of cofilin/twinfilin and the HFD domain from actin, and formation of a complex between ADP-actin and the C-terminal half of CAP for nucleotide exchange^34^. Thus, interplay between the N- and C-terminal domains of CAP can efficiently recycle actin monomers and cofilin for new rounds of filament assembly and disassembly, respectively.

## Discussion

**Fig. 7:**
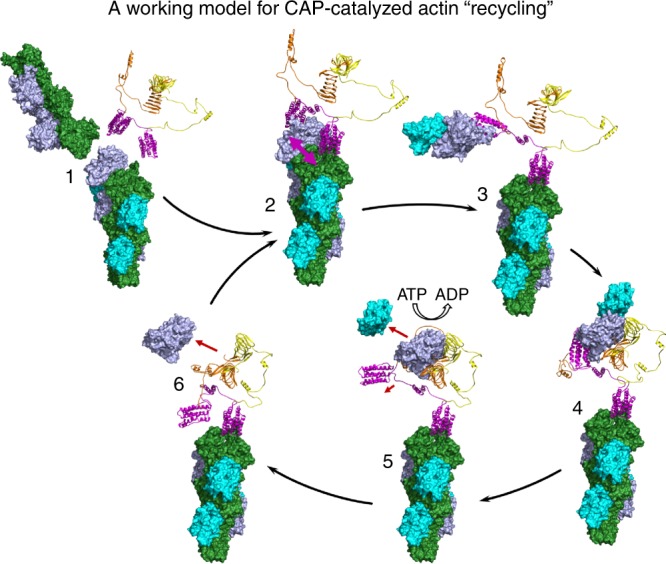
A working model for actin filament depolymerization and monomer recycling by CAP. The different states of the model are based on prior structural data^34,43,44^, as well as crystal structure and atomistic molecular dynamics models from this study. The CARP domain dimer^34^ (yellow/orange) is connected by Rosetta-modeled linkers to the HFD domains (magenta). Please note that for simplicity CAP is presented as a dimer in the model. (1) ADP-actin filaments (blue/green) are severed by cofilin (cyan). (2) CAP binds with high affinity to the cofilin-decorated filament pointed end through its two HFD domains, and destabilizes the interface between the two terminal subunits of the actin filament. (3) The ultimate ADP-actin monomer dissociates from the filament pointed end, due to spatial restrains and lost contacts to the penultimate monomer. The HFD domain and cofilin stay associated with the actin monomer. (4) The freshly depolymerized actin monomer is handed over from the HFD domain to the CARP domain of CAP, while the second HFD domain remains bound to the actin filament pointed end. (5) The WH2 domain of CAP wraps around the actin monomer, and together with the adjacent CARP domain displaces the HFD domain and cofilin from the actin monomer. It subsequently catalyzes ADP-to-ATP nucleotide exchange on actin. (6) The ATP-actin monomer is released from the C-terminal half of CAP, and the free HFD domain binds to the penultimate actin in the filament to begin a new cycle of depolymerization.

Our work identifies CAP as a remarkably efficient actin filament pointed end depolymerizing protein and actin recycling machinery, and elucidates the underlying molecular mechanism. Based on these data, we propose a working model for how cofilin-severed actin filaments are disassembled from their pointed ends by CAP, and how the monomers are recycled for a new round of filament assembly (Fig. 7). Actin filaments are asymmetrically severed at the interface between the bare and cofilin-decorated segments, in a way that most severing events occur towards the pointed end of the cofilin segment. Thus, majority of the resulting pointed ends are cofilin-decorated^11,14,16,47^. We show that CAP binds to the pointed ends of cofilin-decorated actin filaments through its N-terminal HFD domains. Experiments with monomeric and dimeric HFD domains suggest that both terminal subunits of an actin filament must be occupied by HFD domains for efficient destabilization of the filament pointed end, and subsequent depolymerization. As CAPs are larger oligomers^22,48^, this may further increase the depolymerization activity of CAP by allowing the next HFD domain to associate with the newly available subunit of the filament pointed end immediately following the dissociation of the first monomer. The N-terminal HFD domain and the C-terminal CARP domain bind actin monomers through nonoverlapping surfaces, and this allows an efficient delivery of the newly depolymerized actin monomer from the N-terminal half to the C-terminal half of CAP, which promotes ADP-to-ATP nucleotide exchange on actin to re-charge it for a next round of polymerization. Thus, CAP work as a molecular machine that drives two critical steps of the actin filament turnover cycle; filament depolymerization and subsequent monomer recycling (Fig. 7). Our imaging analysis demonstrated that N-CAP binds filament pointed ends with a half-life of ~0.4 s, proposing that it can remove on average ~5 subunits per association when assuming a depolymerization rate of 13 subunits/s (Fig. 2d, g, h). However, further work is required to reveal the processivity of the full-length CAP at the actin filament pointed end.

Earlier studies suggested that CAP, and its N-terminal half, accelerate actin dynamics by filament severing^32,33,37^. In our experiments on single cofilin domains, as well as globally on actin filaments, we did not detect significant increase in filament severing catalyzed by N-CAP (Supplementary Fig. 3c−d). However, a modest increase in severing frequency was observed with the full-length protein. The small increase in filament severing with full-length CAP, which tended to stick on the coverslip surface, may arise from unspecific anchoring of actin filaments to the microchamber by CAP. This would constrain their twist and thereby enhance filament severing^13^. It is important to note that in the previous reports demonstrating the severing activity of CAP, actin filaments were immobilized with multiple anchoring points on the coverslips. This could complicate the observation of new severing events, particularly when the severed fragments are not able to diffuse away. It is also possible that CAPs from different species display small differences in their biochemical activities. Nevertheless, our work provides strong evidence that CAP promotes actin filament disassembly mainly through accelerating the pointed end depolymerization of cofilin-decorated actin filaments.

The function of CAP as an efficient actin filament depolymerizing protein is in line with the observed knockdown and knockout phenotypes in various organisms and cell-types, where depletion of CAP results in an accumulation of filamentous actin and diminished actin turnover rates^23,25,26,29^. CAP is also an abundant protein, present in approximately 1:4 ratio to actin in mammalian cells^23^. Our experiments revealed that full-length CAP can also efficiently depolymerize actin filaments in the presence of ATP-G-actin and profilin/ATP-G-actin complexes, demonstrating that it can indeed drive actin filament depolymerization under actin-filament assembly promoting conditions (Supplementary Fig. 7). Previous genetic work on budding yeast also demonstrated that mutations (Phe162Ala and Tyr163Ala) at the HFD domain interface, which based on our structural and biochemical work is critical for actin-binding and acceleration of actin filament depolymerization, disrupt the function of CAP in vivo^36^. These data, together with earlier genetic and structural work on the C-terminal CARP domain of CAP^34,49^, demonstrate that both rapid actin filament depolymerization and subsequent nucleotide exchange are essential functions of CAP in cells. CAP thus promotes two critical steps of actin turnover cycle, and these two functions appear to be precisely coordinated within this multifunctional protein. In the future, it will be important to reveal the precise structural mechanism and biochemical role underlying the oligomerization of CAP, as well as to reveal how posttranslational modifications regulate the different functions of CAP^31,50^.

This study, together with a related publication^51^, change the current view on actin dynamics according to which actin filament disassembly in cells is predominantly driven by ADF/cofilin-catalyzed filament severing, which increases the number of filament ends that undergo spontaneous depolymerization^52^. Identification of CAP as a protein that catalyzes rapid actin filament pointed end depolymerization also explains why CAP is so critical for actin-dependent cellular and developmental processes in all eukaryotes^23,25,26^.

## Methods

### DNA constructs

All mouse N-CAP proteins, N-CAP-GFP, full-length CAP1, the HFD domain, GST-fusion of the HFD domain and the C-terminal ADF-H domain of twinfilin, were cloned into pSUMOck4 bacterial expression vector (a kind gift from Inari Kursula, University of Bergen, Norway) to express a SUMO-tagged fusion protein which leaves a native N-terminus after cleavage with SENP2 protease. For the CARP domain and C-CAP constructs, we used pCoofy18 bacterial expression vector (a kind gift from Addgene). For details of the protein constructs and primers used for cloning the constructs, see Supplementary Table 2.

### Expression and purification CAP proteins and its fragments

All mouse CAP proteins and its fragments, except for full-length CAP1, were expressed at +22 °C in LB auto-induction media (AIMLB0210, Formedium) for 24 h in BL21(DE3) *E. coli* (from Novagen).

Full-length mouse CAP1 was expressed in LB medium using ArcticExpress (DE3) *E. coli* cells. First, we inoculated a starter culture containing kanamycin (20 µg/ml) and gentamycin (20 µg/ml) that was incubated for 6 h at +37 °C. The 3.6-l main culture, containing only kanamycin (20 µg/ml), was inoculated and grown to OD_600_ of ~0.4 at +37 °C shaking at 240 rpm. The culturing temperature was changed to +13 °C for 1 h prior to protein expression that was induced by addition of 0.26 mM IPTG for 46 h. All bacterial pellets were collected by centrifugation, resuspended in 50 mM Tris-HCl, 150 mM NaCl, 25 mM imidazole, pH 7.5, snap-frozen with liquid N_2_ and stored at −80 °C.

All CAP proteins and the ADF-H domain of twinfilin were purified using a similar workflow. First, bacteria were lysed by sonication in the presence of lysozyme (0.5 mg/ml), DNAse (0.1 mg/ml) and protease inhibitors (200 µg/ml PMSF, 1 µg/ml leupeptin, 1 µg/ml aprotinin, 1 µg/ml pepstatin A; all from Sigma-Aldrich), and lysate was clarified by centrifugation. Supernatant was loaded into a 1 ml HisTrap HP Ni-NTA column (GE Healthcare) and washed extensively (>20 column volumes) with 50 mM Tris-HCl, 150 mM NaCl, 25 mM imidazole, pH 7.5. Protein was eluted by 25–250 mM imidazole gradient on AKTA Pure machine (GE Healthcare). Peak fractions were pooled and SENP2 protease was added to final concentration of 40 µg/ml for removal of the SUMO-tag. Mixture was dialyzed O/N at 4 °C using SnakeSkin dialysis tubing in 1 l of 20 mM HEPES, 300 mM NaCl, 2 mM DTT, pH 7.4 buffer. Next day, the cleaved SUMO-tags were removed with Ni-NTA agarose beads (Qiagen) in cases where the SUMO-tag and cleaved protein were equal in size. Proteins were concentrated with Amicon Ultra-4 10 kDa centrifugal filter (Merck) and loaded into HiLoad 16/600 Superdex 200 gel filtration column (GE Healthcare) equilibrated in 5 mM HEPES, 100 mM NaCl, 1 mM DTT, pH 7.4. Peak fractions were collected, concentrated, and frozen by snap-freezing in N_2_ for −80 °C storage.

The full-length CAP was purified with following exceptions. We used a 5 ml HisTrap HP Ni-NTA column instead of 1 ml column. After dialysis and gel filtration with HiLoad 16/60 Superdex 200 gel filtration column, the major peak fractions were run through Superose 6 increase 10/300 GL gel filtration column to gain better separation of the mixture of oligomeric states. Finally, the major peaks from several runs were combined, concentrated at 5-min spin-intervals using Amicon Ultra-4 30 kDa centrifugal filter, and stored as above. CARP and C-CAP proteins were purified as above with the exception of usage of 3C protease (0.01 mg/ml) for tag cleavage.

### Other proteins

Rabbit muscle actin, labeled actins, profilin, mouse cofilin-1, capping protein, and biotin-gelsolin were prepared as described in ref. ^16^. ADP-actin was prepared as described in ref. ^34^. Briefly, 30 µM ATP-G-actin solution containing 0.3 mM glucose and 1 unit/ml of hexokinase (Sigma) was dialyzed against nucleotide exchange buffer (5 mM Tris-HCl, 0.1 mM MgCl_2_, 0.05 mM EGTA, 0.2 mM ADP, 1 mM DTT, pH 8.0) for 4 h at 4 °C. All experiments were performed using rabbit muscle α-actin.

### Actin pointed end depolymerization assays

Measurement of the actin filament pointed end depolymerization rate was performed using a microfluidics device paired to a microscopy setup, as described^16^. In short, we prepared a chamber with poly dimethyl siloxane (PDMS, Sylgard), which was mounted on a cleaned coverslip. Microfluidic chambers were 20 µm in height. The three inlets and the outlet were connected to pressure-controlled tubes filled with solutions of different biochemical composition (Fluigent microfluidics device).

After mounting, chambers were rinsed with dH_2_0 and F-buffer (5 mM HEPES pH 7.4, 100 mM KCl, 1 mM MgCl_2_, 0.2 mM EGTA, 0.4 mM CaCl_2_, 0.2 mM ATP, 10 mM DTT and 1 mM DABCO). The chamber was then successively exposed to: 150 µl of 0.1% biotin-BSA in F-buffer; 300 µl of 5% BSA; 150 µl of 3 µg/ml neutravidin in F-buffer; and 10–100 µl of 1–3 pM biotin-gelsolin. Prior to experiments, actin filaments were polymerized (8 µM, >30 min) in F-buffer. A fraction of actin monomers was labeled with Alexa-488 or 568. Filaments were finally injected into the chamber and anchored to the coverslip by the gelsolins until desired density.

To measure the depolymerization rate of cofilin-decorated pointed ends, filaments were first saturated with 2 µM cofilin, and exposed to 2 µM cofilin supplemented with different CAP proteins. The depolymerization rate was analyzed with ImageJ, first making a kymograph for each filament (function reslice) and manually fitting a slope along the pointed end. Filaments, which pointed ends could not be clearly tracked or had possible (un)observed pauses^53^ or severing events were discarded from analysis.

To minimize the effect of protein label on our measurements, we used either 10% or 16% labeling fraction of actin depending on the microscopy setup. We did not observe effects of labeling fraction for N-CAP activity in our experiments. All experiments were performed at room temperature, in non-temperature-controlled setup.

### Reannealing of actin filaments

Preformed, steady-state F-actin solutions (in F-buffer) with different fluorescent labels were mixed and incubated in order to quantify reannealing. The mixed solution contained 4 µM of Alexa568-actin (10% labeled) and 4 µM of Alexa488-actin (10% labeled), with or without N-CAP and mutants. After 4 min at room temperature, the mixed F-actin solution was diluted 100× in buffer supplemented with 0.2% methylcellulose, and flowed into an open chamber made with double-sided tape sandwiched between two coverlips, and passivated with BSA. Images series (2 s interval) were acquired in at least three different fields of view. The number of green (Alexa488) F-actin segments were counted, as well as the number of these segments that were connected to a red (Alexa568) F-actin segment, in order to determine the ratio of two color segments plotted in Fig. 2f. Monitoring the diffusion of the filaments allowed us to determine unambiguously when two segments were connected. Controls (with no N-CAP in the F-actin mix) were repeated several times, and experiments (with N-CAP or mutants) at least twice.

### Actin severing assays

To investigate the impact of N-CAP on the severing by cofilin, we used two different methods, either (1) by measuring the fraction of single cofilin domains that have not yet led to a severing or by (2) quantifying the global number of severing events per µm of actin filament.

(1) Severing associated with single cofilin domains (Supplementary Fig. 3c). We followed the procedure described previously^16^. Briefly, inside a microfluidic chamber, 12% Alexa-488-labeled actin filaments were polymerized from spectrin-actin seeds, anchored nonspecifically to a BSA-passivated coverslip. Filaments were aged for 15 min with a solution of G-actin at critical concentration (100 nM G-actin), so that filaments become >99% ADP^46^. Filaments were then continuously exposed to 500 nM mCherry-cofilin-1 alone, with 2 µM full-length CAP1 or with 10 µM N-CAP. On ImageJ, kymographs of the filaments were then constructed to follow the nucleation and assembly of single cofilin domains and severing events at the interface with bare actin segments.

For each domain, time 0 was defined at the frame on which they nucleated. We then recorded either the time at which they induced a severing event, or when they are lost due to severing by another domain, merging, or censoring event. The fraction of domains that have not induced a severing event was then calculated using a classical Kaplan−Meier method.

(2) Total number of severing events/µm (Supplementary Fig. 3d). Inside flow chambers (between two coverslips spaced by double-sided tape, we first injected prepolymerized Alexa-488-labeled filaments (in F-buffer, with 0.2–0.3% Methylcellulose). The solution was then exchanged with 500 nM unlabeled cofilin-1 and 0, 1 or 10 µM NCAP. After the exchange, filaments that remained near the surface were analyzed as follows.

The number of severing events per µm was calculated as the cumulative function

1 $$f_{(t)} = \mathop {\sum}\limits_{u = 0}^t {\frac{{N_{{\mathrm{{sev}}}(u)}}}{{\mathop {\sum }\nolimits_i l_{i(u)}}}},$$

where *N*_sev(*u*)_ is the number of severing events at time *u*, and $\mathop {\sum}\nolimits_i {l_{i(u)}}$ is the sum over all filaments *i* of their length at time *u*. As the solution does not contain G-actin, the filaments depolymerize, and the filament length thus decreases over time.

### Binding of N-CAP to filaments pointed ends

The experiment was performed in flow chambers (see Actin severing assay (2)), passivated with biotin-PLL-PEG and functionalized with neutravidin. F-actin (10% Alexa-568, 1% biotin) was polymerized overnight at 4 µM. Just before injection into the chamber, F-actin was diluted down to 200 nM and mixed with 100 nM N-CAP-GFP, 2 µM cofilin-1, and 4 nM capping protein, in F-buffer supplemented with 0.2% methylcellulose. Images of actin filaments were acquired before and after a stream acquisition of the GFP channel (5 frames/second over 12 s, TIRF microscopy). For analysis of binding to the filament ends, actin filaments that did not move during the stream acquisition were blindly selected. The fluorescence was measured on the two ends of each filament, on a 3 by 3 pixels area. A binding event was detected when the fluorescence would go over an arbitrary threshold (same value for all filaments and filament ends). As capping protein should protect the barbed end, the pointed end was attributed to the one with the most binding events. N-CAP-GFP was detected in 17% of the data points at the filament pointed end, while unspecific association of N-CAP-GFP at the vicinity of filament barbed end was detected in 1.7% of the data points. The survival fraction of N-CAP at the pointed end was then calculated and fitted with a single exponential to measure the unbinding rate.

### Crystallization and structure determination

The mouse HFD domain was crystallized with 10×His-tag present, and purified as described above, with exception of using 5 mM HEPES, 50 mM NaCl, 0.2 mM DTT, 0.01% NaN_3_, pH 7.5 buffer in gel filtration. Sample was concentrated to 7–10 mg/ml prior to crystallization and mixed 1:1 to 0.1 M sodium cacodylate, 12% PEG4000 (w/v), pH 6.1 in using a sitting drop method with drop size of 200 nl in 96-well format. After 2 weeks of incubation large needle-like crystals were observed. For remote data collection at Diamond Light Source (UK, Didgot) at beamline I03 the crystals were cryo-protected by soaking in mother liquid containing 25% glycerol and snap-frozen in N_2_ for shipping to the beamline. The data were collected at 100 K using 0.9763 Å wavelength, Pilatus3 6M detector, 30% transmission power, 0.05 s exposure and 0.1° oscillation angle as a total of 2400 frames. The diffraction data were integrated and scaled with X-ray Detector Software (XDS)^54^. The initial solution was obtained with molecular replacement using PHASER^55^ and PDB = 1s0p as a search model. A solution with four HFD domains present in an asymmetric unit was found, after which multiple rounds of refinement with BUSTER^56^ and manual building in COOT^57^ yielded a good fit to the data (see Table 1). Further improvement was obtained by refining the data with the introduction of translation-liberation-screw parameters (1/chain), removal of noncrystallographic restrains and individual atomic B-factor modeling. The final *R*_work_/*R*_free_ for the model was 18.6%/23.0% with good overall geometry.

For crystallization of the tripartite complex (of ADP-actin, HFD domain of mouse CAP1, and C-terminal ADF-H domain of mouse twinfilin-1), ADP-actin was prepared in O/N dialysis at +4 °C in 5 mM HEPES, 0.2 mM MgCl_2_, 0.2 mM ADP, 0.2 mM EGTA, 0.3 mM glucose, 0.5 mM β-mercaptoethanol, pH 8.0. Actin solution, containing 0.3 mM glucose and 5 U/ml of hexokinase, was transferred to a Slide-A-Lyser dialysis membrane and put on a floater device at +4 °C. The next day before complex formation, actin was centrifuged for 20 min at 355,040 × *g* with TLA-120 rotor. Proteins were mixed in 1:1.1:1.1 ratio (actin, HFD domain, ADF-H domain), concentrated to 10–20 mg/ml and used for crystallization as above. Hits were obtained from several different conditions, and the best diffracting crystal was obtained at ~10 mg/ml concentration of the complex mixed 1:1 in 0.1 M HEPES, 0.1 mM KCl, 10% PEG4000 (w/v), pH 7.0 with sitting drop size of 200 nl. On the first day, several small diamond-shaped crystals appeared in the drop. On the third day, a large rod-like crystal appeared while all the small crystals were dissolved. For remote data collection at Diamond Light Source (UK, Didgot) at beamline I03, the crystals were cryo-protected by soaking in LV CryoOil (MiTeGen) and snap-frozen in N_2_ for shipping. The data were collected at 100 K using 0.9762 Å wavelength, Pilatus3 6M detector, 20% transmission power, 0.05 s exposure and 0.15° oscillation angle as a total of 2400 frames. The diffraction data were integrated and scaled with XDS^54^. An initial solution was obtained with molecular replacement using PHASER^55^ and PDB = 3daw as a search model that showed clear extra density in the pointed end of actin monomer. Thus another molecular replacement was carried out, and an initial model for the tripartite complex was obtained using BALBES^58^ with 3daw and 1s0p as search models. This yielded a solution with *Q* = 0.787 and *R*_work_/*R*_free_ = 29.7%/35.6%. The asymmetric unit contained a single 1:1:1 complex of HFD:ADF-H:ADP-actin. Multiple rounds of refinement with BUSTER^56^ and manual building in COOT^57^, especially rebuilding of D-loop and connecting loops of α-helices in the HFD domain were required to improve the model. Finally, addition of waters, introduction of translation-libration-screw parameters (1/chain), and individual atomic B-factor modeling yielded a model with final *R*_work_/*R*_free_ of 16.6%/19.4% with good overall geometry.

### Examining protein−protein interactions by gel filtration

For studying actin monomer binding, ADP-G-actin was prepared as described in ref. ^34^. All gel filtration experiments were performed at +4 °C with 0.5 ml/min running speed and 0.5 ml fractionation with Superdex 200 increase 10/300 GL gel filtration column equilibrated in 5 mM HEPES, 100 mM NaCl, 0.1 mM ADP, 0.1 mM MgCl_2_, 1 mM DTT, pH 7.4. One hundred microliters of complex containing 18 µM ADF-H domain of twinfilin, 15 µM other proteins was injected to the column and analyzed for elution. Peak fractions were also analyzed by SDS-PAGE. Actin monomer competition experiments were performed as above, by including 15 µM C-CAP or CARP domain to the samples. Peak fractions were analyzed on SDS-PAGE, gels were imaged with ChemiDoc XRS + imaging system (Bio-Rad), and quantified using Image Lab (Bio-Rad).

### Native-PAGE

Mini-Protean TGX 10% gels (Bio-Rad) were pre-run in cooled running buffer (25 mM Tris, 195 mM glycine, 0.5 mM ADP, 0.1 mM MgCl_2_, pH 8.5) for 1 h before loading. Samples were prepared in ADP-actin dialysis buffer (5 mM HEPES or Tris, 0.1 mM MgCl_2_, 0.1 mM EGTA, 0.2 mM ADP, 0.3 mM glucose, 0.1 mM DTT, pH 8.0) at 20 µM concentration of each protein, mixed at 1:1 ratio with 2× loading buffer (running buffer containing 20% glycerol, bromophenol blue, no ADP or MgCl_2_) and then applying 5 µl volume to 50 µl sample wells. Gels were run at 100 V on ice for 4 h.

### Fluorometric actin filament disassembly assay

The steady-state disassembly of ADP-actin filaments was performed as following: 5% pyrene-actin was polymerized in 20 mM HEPES, 100 mM NaCl, 1 mM MgCl_2_, 1 mM ATP, 1 mM DTT, pH 7.4 for 60 min. The filament barbed ends were capped by 50 nM capping protein. Subsequently, 0.5 µM cofilin (and 0.5 µM N-CAP) were added, and the reaction was started by adding 4 µM Vitamin D binding protein (monomer sequestering agent). Final concentration of actin was 2.5 µM. Actin disassembly was measured by following pyrene fluorescence with excitation at 365 nm and emission at 407 nm on fluorescence spectrophotometer (Agilent) for 2400 s at 22 °C.

### CD spectrometry

The CD spectra for the N-CAP wild type, mutants and the HFD domain were measured at 20 °C with the J-720 spectropolarimeter (Jasco), in 300 µl quartz cuvette of 0.1 cm light path length with the following parameters: continues scanning mode with scanning speed of 50 nm/min, bandwidth 0.5 nm, wave range 190–260 nm, data pitch 0.5 nm. All the proteins were diluted to 15 µM with 10% PBS buffer. Accumulation of ten scans for each protein has been plotted as a single curve.

### Models for atomistic MD simulations

The cofilin-decorated actin models were based on the 3.8 Å resolution cryo-electron microscopy structure (PDBID: 5YU8)^41^. The missing loops were built using RosettaCM^59^ and RosettaScripts^60^ in the presence of the electron density map^41^ imposing the associated helical symmetry. To match the experimental constructs in this work, actin and cofilin sequences were, in this stage, changed from chicken’s to rabbit’s and mouse’s, respectively. Over 1000 models were generated. They were, then, sorted based on their total score and the ones that contained structural artifacts (e.g., *cis* peptide bonds) were filtered out. Molecular dynamics (MD) simulations were initiated from the top scoring three models (Supplementary Table 1, simulations F1−3).

The HFD-actin complex was isolated from the crystallized HFD domain/actin/twinfilin ADF-H domain complex and separately docked on to the selected cofilin-decorated actin filament models described above. For this purpose, the isolated HFD-actin was superposed onto each pointed end actin monomer, which was then replaced with the HFD-actin. This way, the crystal structure conformation of actin-HFD dimer is transferred to the tip of the cofilin-decorated actin. The docked models were first locally refined using the docking protocol^61^ followed by restrained relaxation with the fast relax^62^ protocol distributed with the Rosetta Software Suite. This process was applied separately for each cofilin-decorated actin filament selected for MD simulations (Supplementary Table 1, simulations C1−3).

### Construction of the pointed end segment for MD simulations

Each simulation was performed on a pointed end segment of the selected cofilin-decorated actin filament model, which was created by slicing the model perpendicular to the axis of the filament. The plane of the slicing was chosen so that four whole ADP-Mg^2+^ bound actin monomers, and three whole cofilin monomers were included within the segment (Supplementary Table 1, simulations F1−3). The segment also contained the two HFD domains at the pointed end in the HFD bound systems (Supplementary Table 1, simulations C1−3). The pointed end segment also contained several polypeptide fragments from cofilins and actins at its barbed end (Supplementary Fig. 5a). To maintain their conformation and position during the simulations, these broken chains were kept positionally restrained throughout the simulations as follows: A layer of residues at the interface with the rest of the pointed end segment were left free; all heavy atoms near the plane of slicing and only backbone heavy-atoms for the regions in between were restrained with a force constant of 100 kJ/mol/nm^2^ (Supplementary Fig. 5a). These partially restrained polypeptide fragments at the barbed end act as a platform during the simulations. This approach maintains both the filament-like protein−protein interface at the barbed end and the orientation of the filament during the simulations.

The protonation states of residues were determined at the neutral pH based on p*K*_a_ calculations using PROPKA3^63^. Each actin H73 residue was methylated (*N*_τ_-Methyl-l-histidine, HIC), and the N-termini of actin, cofilin, and HFD were acetylated. The topologies for each molecule in the systems was prepared with the LeAP program distributed with ambertools18^64^, which were then converted to the GROMACS format using the ParmEd tool.

Each pointed end slice created from the selected models was placed in a hexagonal prism simulation box with its long-axis aligned with the *z*-axis. The box dimensions were chosen to have a minimum distance of about 17 Å between the filament and each face of the box (Supplementary Table 1). Each system was solvated with 0.15 M NaCl solution with the numbers of ions adjusted to neutralize the system.

Before production runs, steepest descent minimization and successive short equilibration simulations (in total ~7 ns) in the NVT and NpT ensembles using the Berendsen thermostat and barostat^65^ were performed. Harmonic positional restraints were applied to the pointed end slice during these equilibration simulations. A smaller group of atoms (all heavy-atoms, the protein backbone, and finally only the C_α_ atoms) were restrained in each consecutive equilibration simulation with a force constant of 1000 kJ/mol/nm^2^.

The systems that were branched from the others (Supplementary Table 1; C1′, C2′. F2′) were prepared by making the suitable modification (docking or removing HFD domains), removing the overlapping solvent molecules (when HFD domains were docked), and readjusting the box size and solvent atoms for the new system size. Staged NVT and NpT equilibration with restraints were performed as mentioned above (in total ~200 ps for simulations where HFD domains were removed, and in total ~4 ns where it is docked).

All production runs were performed for 1–2.5 μs in the NpT ensemble with only the aforementioned positional restraints on the platform region.

### Force fields and parameters in MD simulations

The following force fields and parameter sets were employed in the MD simulations: Amber ff14sb^66^ for the proteins, TIP3P model^67^ for water, the monovalent ion parameter set by Joung and Cheatham^68^ for Na^+^ and Cl^−^, an octahedral multisite ion model by Saxena and Sept^69^ for Mg^2+^, and a polyphosphorylated compound parameter set by Meagher et al.^70^ for ADP. Missing bonded parameters for methyl-histidine (*N*_τ_-Methyl-l-histidine, HIC) were adopted from the GAFF2 force field^64^. The atomic charges were calculated following the protocol by Duan et al.^71^ R.E.D.III.5 software^72^ was employed for multiconformation restrained electrostatic potential (RESP) fitting using an extended and an α‐helical conformations of HIC dipeptide (Ace‐HIC‐Nme). All quantum-chemical calculations were performed using the Gaussian09 program suite^73^ at the b3LYP/cc-pVTZ level of theory. Charge calculations were performed with the IEFPCM model in a polarizable continuum with a dielectric constant of 4 by setting the solvent as ether.

### MD simulation protocols

All simulations were carried out using GROMACS 2018 ^74^. The equations of motion were integrated using a leap-frog algorithm with a 2 fs time step. All bonds were constrained using the LINCS algorithm^75^. The simulation protocols were chosen according to ref. ^66^. Long-range electrostatic interactions were treated by the smooth particle mesh Ewald scheme^76^ with a real-space cutoff of 0.8 nm, a Fourier spacing of 0.12 nm, and a fourth-order interpolation. Lennard–Jones potential with a cutoff of 0.8 nm was used for van der Waals interactions. Long-range dispersion corrections were applied for energy and pressure^77^.

All production simulations were performed in the NpT ensemble. The pointed end slice, the platform, and solvent (water and 0.15 M NaCl) were coupled to separate temperature baths at 310 °K using the Nosé−Hoover thermostat^78,79^ with a time constant of 1.0 ps. Isotropic pressure coupling was performed using the Parrinello–Rahman barostat^80^ with a reference pressure of 1 atm, a time constant of 5 ps, and a compressibility of 4.5 × 10^−5^ bar^−1^.

### Analysis of the MD simulations

All analyses were performed using VMD^81^ and in-house scripts. The MMGBSA calculations were performed using the MMPBSA.py software^82^ distributed with ambertools18^64^ employing the modified GB model developed by Onufriev et al.^83^ with 0.15 M salt concentration (frames sampled every 100 ns discarding the first 500 ns of each simulation).

### Structural analyses

For analysis of different actin structures and their conformational states presented in Fig. 1d, we followed the protocol by Tanaka *et al*^41,84^. using the F-actin structure (PDB 6djo) as the reference.

### Statistical analysis and reproducibility

Data in Figs. 2d, 4b and 4d were pooled from several experiments performed on different days, thus representing data from several independent experiments. Panels 2f, 2g, 2h and 3d present data analyzed from a single representative experiment. *n* describes number of filaments analyzed for panels 2d, 2f, 2g, 4b and 4d. *n* in panel 6c corresponds to number of times the experiment was conducted.

Experiments in Supplementary Figs. 2a−d, 3c, 6a−d were performed with similar results at least twice. Experiments in 3d, the CARP domain competition experiment in 6a, and the experiments under assembly promoting conditions in Fig. 7 were conducted once.

### Reporting summary

Further information on research design is available in the Nature Research Reporting Summary linked to this article.

## Supplementary information

Supplementary Information

Supplementary Movie 1

Reporting Summary

Description of Additional Supplementary Files

Peer Review File

## Data Availability

Data supporting the findings of this manuscript are available from the corresponding author upon reasonable request. A reporting summary for this Article is available as a Supplementary Information file. The crystallographic data are stored in Protein Data Bank under accession codes 6RSQ and 6RSW. The molecular dynamics simulation data are available from Zenodo (10.5281/zenodo.3340994). The source data underlying Figs. 1d, 2d, 2f, 2h, 3d, 4b, 4d, 5d, 6c and Supplementary Figs. 2, 3c, d, 6 and 7 are provided as a Source Data file.

## Source Data

Source Data
