# Supplementary material for: Mechanism of synergistic actin filament pointed end depolymerization by cyclase-associated protein and cofilin
Source: Nat Commun. 2019 Nov 22;10:5320. doi: 10.1038/s41467-019-13213-2 (PMC6876575; doi:10.1038/s41467-019-13213-2)
Supplement: Supplementary file 1 — Supplementary Information [file 41467_2019_13213_MOESM1_ESM.pdf]

Kotila *et al.* (2019) Supplementary Information

SUPPLEMENTARY FIGURES

Supplementary Fig. 1

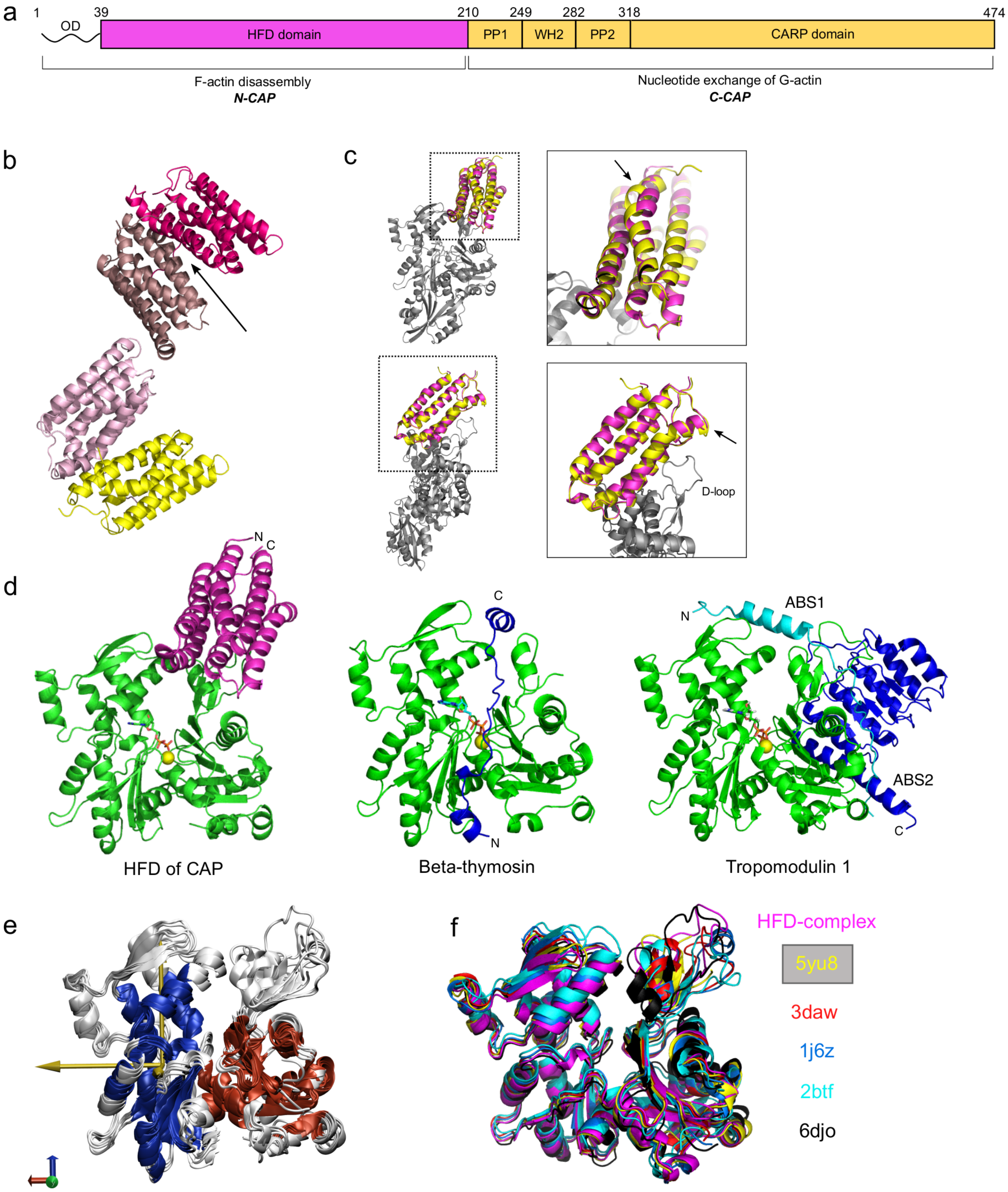

Supplementary Figure 1. The crystal structure of the HFD domain of mouse CAP1. (a) The domain structure of CAP divided into its functional units. The N-terminal half of CAP (N-CAP), composed of OD and HFD domains, accelerates actin filament disassembly and binds ADF/cofilin-actin monomers complexes[1–4]. The C-terminal half of CAP (C-CAP) binds ADP-actin monomers and catalyses ADP-to-ATP nucleotide exchange on actin monomers[5]. Abbreviations: OD=oligomerisation domain, HFD=helical folded domain, PP1=polyproline region 1, WH2=Wiskott-Aldrich homology 2, PP2=polyproline region 2, CARP=CAP and retinitis pigmentosa 2 domain. (**b**) The 2.37 Å crystal structure of the HFD domain of mouse CAP1 (in the absence of actin). The asymmetric unit contained four HFD domains, which make two dimers through a disulphide bond indicated with an arrow (Cys92-Cys92). (**c**) Superimposition of the crystal structures of the HFD domain in the presence (magenta) and absence of actin (yellow). Upper panel: Arrow indicates a kink in the α-helix, which is caused by the disulphide-bond formation in HFD domain crystallized alone. Lower panel: A small movement in the helix of the HFD domain facing actin subdomain 2 was observed, most likely due to formed contacts with the D-loop of actin (indicated with an arrow). **(d)** Comparison of the actin-binding interfaces of the HFD domain and other proteins binding to the pointed end (subdomains 2 and 4) of actin. Actin monomer sequestering protein β-thymosin (PDB=4pl7) binds to both the barbed and pointed end of actin monomers. Actin filament pointed end capping protein, tropomodulin, uses two actin binding sites to associate with the pointed ends of actin filaments (PDB=4pkg, 4pki). (**e**) The analysis of actin conformational states from selected structures. Regions indicated in blue and red present the rigid bodies to define planes for calculating the twist of the outer domain relative to F-actin conformation (6djo). (**f**) Full presentations of the structures shown in Fig. 1D. Corresponding PDB-codes are color-coded and indicated on left.

Supplementary Fig. 2

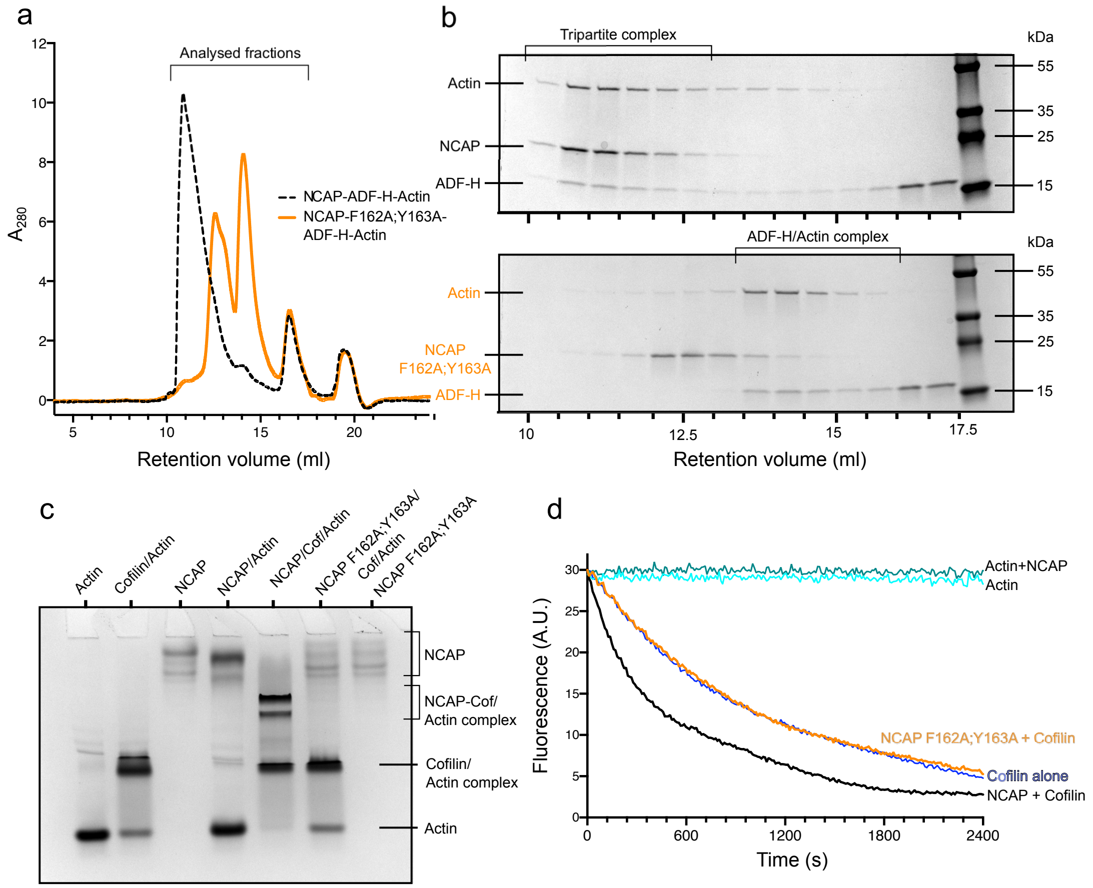

Supplementary Figure 2. Analysis of an N-CAP mutant for actin monomer-binding and filament disassembly. (**a**) Actin monomer-binding was measured by gel filtration for wild-type N-CAP, and for N-CAP containing mutations (F162A and Y163A) in the actin-binding interface 1. The assay was carried out at physiological salt, in the presence of twinfilin's C-terminal ADF-H domain (18 µM) and ADP-G-actin (15 µM). The concentration of wild-type/mutant N-CAP was 15 µM. 100 µl of sample was injected to the column. (**b**) Analysis of the gel filtration fractions from (A) by SDS-PAGE. **(c)** Native-PAGE analysis of N-CAP binding to ADP-G-actin/cofilin-1 complexes. Samples contained 10 µM each protein, and 5 µl aliquots were loaded on the gel. Actin forms a complex with cofilin-1. N-CAP does not efficiently bind actin monomers alone, but associates with the actin monomer/cofilin-1 complex. The F162A, Y163A mutant N-CAP does not associate with the actin monomer/cofilin-1 complex. Please note that cofilin-1 does not enter the gel due to its high isoelectric point. (**d**) Fluorometric actin filament disassembly assay carried out with 2.5 µM F-actin (5% pyrene-labelled) in the presence of 50 nM capping protein and 4 µM Vitamin D binding protein (actin monomer sequestering agent). Actin filament disassembly was measured with indicated proteins at 0.5 µM. Please note that cofilin-1 increases the disassembly of actin filaments, and this can be augmented by wild-type N-CAP, but not by the F162A,Y163A mutant N-CAP.

Supplementary Fig. 3

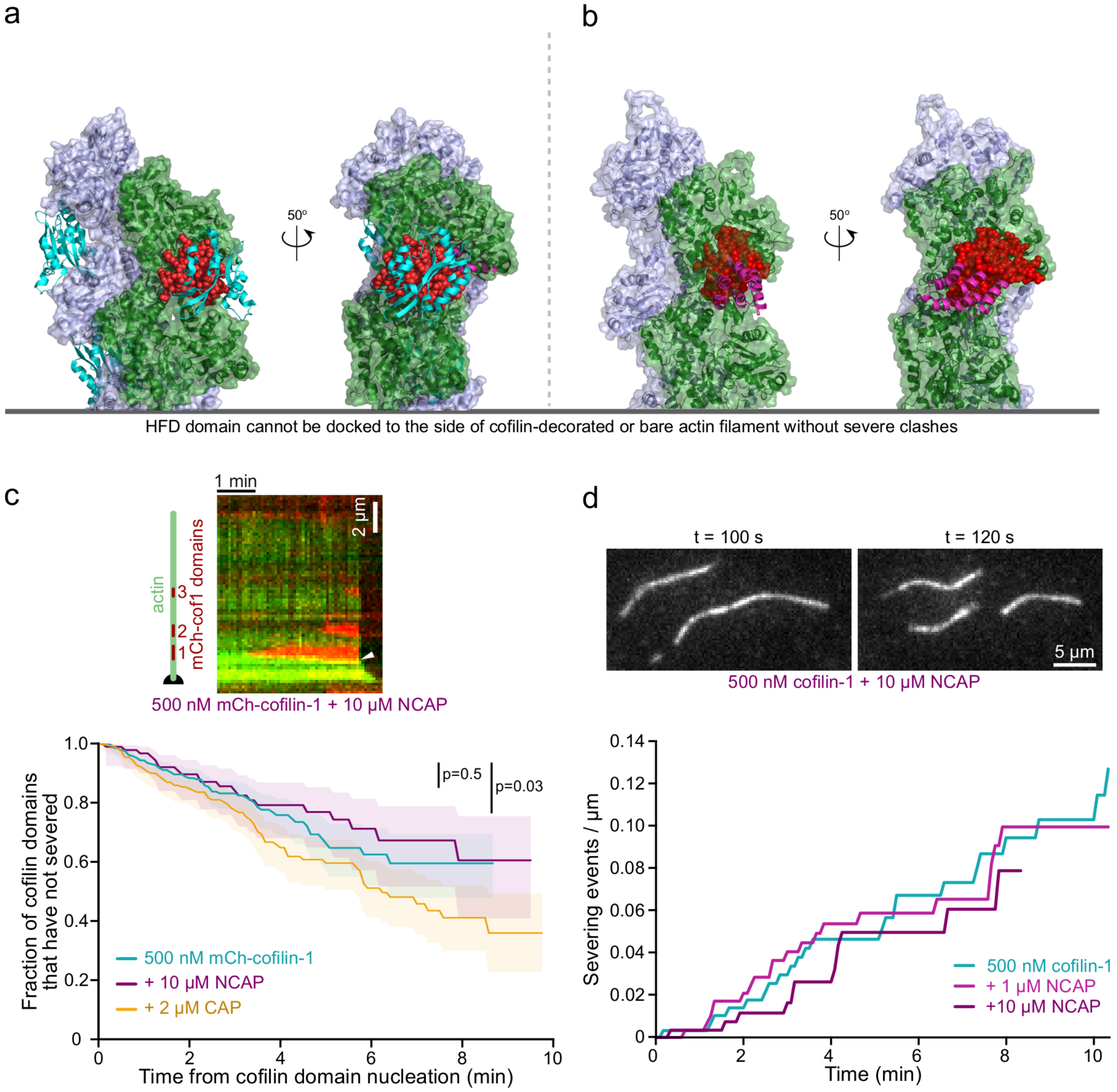

Supplementary Figure 3. The HFD domain cannot bind to the side of an actin filament and does not induce filament severing. (a) The binding interface of the HFD domain on actin overlaps with the one of cofilin and with the barbed end of the next actin monomer in the cofilin-decorated actin filament. Clashes are indicated with red spheres. (**b**) The binding interface of the HFD domain overlaps with the barbed end of the next actin monomer. Clashes are indicated with red spheres. **(c)** Severing measured at single mCherry-cofilin-1 domain boundaries. Top: A typical kymograph of a filament exposed to 500 nM mCherry- cofilin-1 and 10 µM N-CAP. Arrowhead indicates the severing event at the boundary of a domain (numbers). Bottom: fraction of mCherry-cofilin-1 domains that have not induced a severing event, versus time. Filaments were constantly exposed to 500 nM cofilin, alone, with 2 µM full-length CAP or with 10 µM N-CAP. The time 0 is defined for every single cofilin domain at the frame on which it appears. Curves were calculated using a Kaplan-Meier method, n = 100 filaments / 361 domains / 66 severing events (500 nM cofilin-1); n = 99 filaments / 243 domains / 76 severing events (+ 2 µM CAP);

40 filaments / 91 domains / 21 severing events (+10 µM N-CAP). P-value: log-rank test (calculated with Python plugin Lifelines). Shaded area: 95% confidence interval. **(d)** Severing measured along full actin filaments. Top: example of a severing event on a filament exposed to 500 nM unlabeled cofilin-1 and 10 µM N-CAP. Bottom: cumulative function of the number of severing events per µm of actin filament vs. time. Pre-polymerized filaments were exposed to 500 nM cofilin-1 (blue, N = 23 filaments, total initial length L_0 = 312 µm) supplemented with 1 µM N-CAP (light purple, N = 23 filaments, L_0 = 322 µm) or 10 µM N-CAP (dark purple, N = 16 filaments, L_0 = 302 µm).

Supplementary Fig. 4

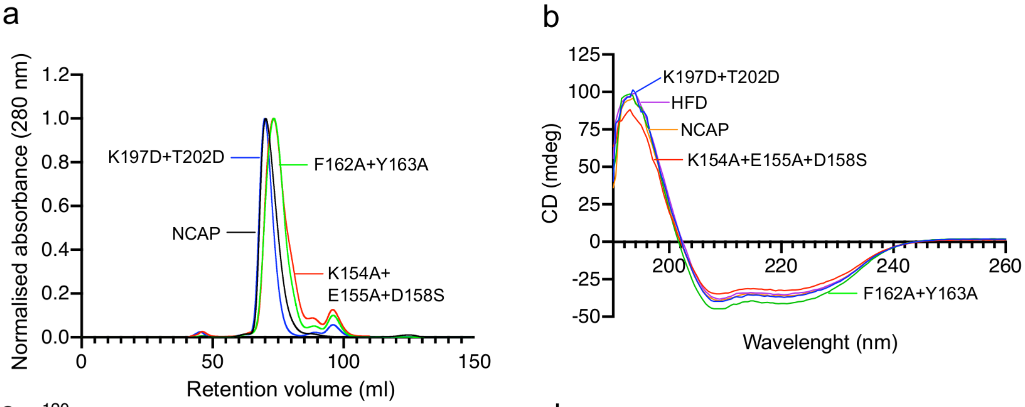

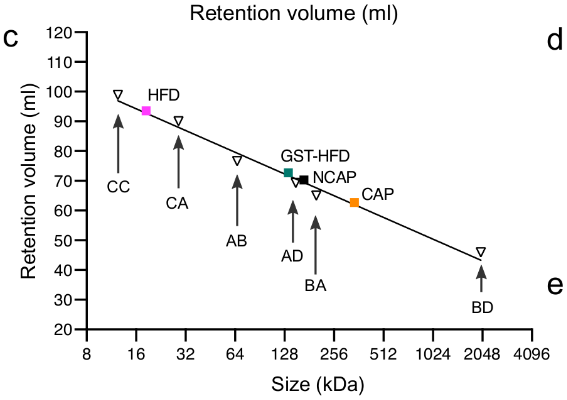

d

Protein	Theoritical size of monomer (kDa)	Calculated size from gel filtration (kDa)
HFD	19.3	18.4
GST-HFD	47.0	135
NCAP	23.2	168
CAP	51.4	341

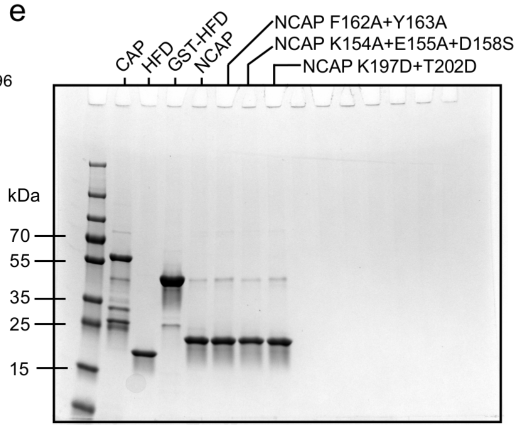

Supplementary Figure 4. Characterization of CAP proteins used in the study. (**a**) Mutant versions of N-CAP eluted in similar volumes in the Superdex 200 HiLoad 16/60 gel filtration column, demonstrating that these mutations do not affect the oligomerization properties of the protein. (**b**) Circular dichroism spectroscopy analysis of wild-type and mutant N-CAP demonstrates that all proteins display similar α- helical fold profile. (**c**) Analysis of the oligomeric state of different CAP proteins used in the study by gel filtration on a Superdex 200 HiLoad 16/60 column. The elution profiles of protein standards (CC=cytochrome C, CA=carbonic anhydrase, AB=albumin, AD=alcohol dehydrogenase, BA=b-amylase and BD=blue dextran) were analyzed and fitted with semi-logarithmic curve. The elution volumes of full-length CAP and it fragments are shown above the line. (**d**) The elution profiles of CAP fragments were plotted and compared to their theoretical monomer sizes. The isolated HFD domain is monomeric, and the GST-HFD construct is most likely an extended dimer. N-CAP and full-length CAP are most likely hexamers based on this analysis. (**e**) SDS-PAGE analysis presenting the purity of the CAP proteins used in this study.

Supplementary Fig. 5

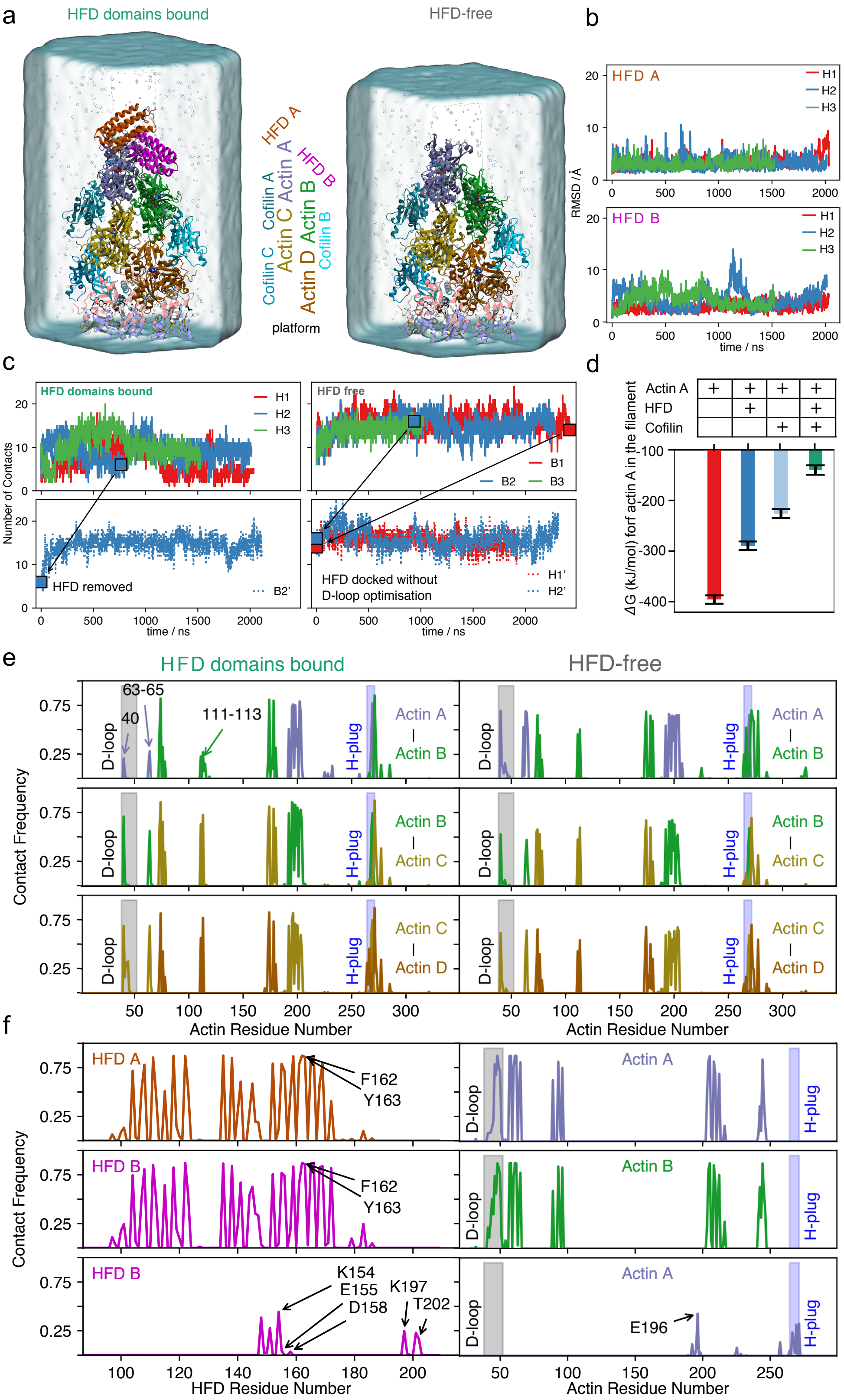

Supplementary Figure 5. The simulation systems and the stability of the filament. (**a**) The simulation systems composed of the cofilin-decorated actin filament (4 actin chains, 3 cofilin chains, and the "platform" region) either with HFD domains bound to the pointed end (HFD-bound, left) or without HFD domains (HFD-free, right). The water slab is shown in transparent blue surface representation. Each protein chain is colored differently with the coloring and naming scheme shown in the central column. The "platform region" is colored according to the type of restraints applied: pink, no restraints; white, only backbone restraints; and blue, heavy atom restraints. The ADP and Mg^{2+} molecules are shown in van der Waals representation. (**b**) The root-mean square deviation (RMSD) of the HFD domain in the MD simulations with respect to that seen in the crystal structure reflecting the stability of the binding mode. RMSDs are calculated with respect to the crystal structure coordinates of the HFD domain after superposing the inner and outer domains of actin A (left panel) and B (right panel) in the simulations onto those of the crystal structure. (**c**) Removing the HFD domains (bottom left) or docking HFD domains to the pointed end without optimizing the D-loop contacts (bottom right) negates the reduction of contacts between actin A and B. The upper panels (Upper left: HFD-bound, and upper right: HFD-free filaments) present the points where control simulations (bottom panel) were branched. (**d**) MMGBSA binding affinities calculated for the pointed end actin A. The affinities for actin alone, HFD-actin pair, cofilin-actin pair, and HFD-actin-cofilin triple are shown. The calculations were performed on frames sampled every 100 ns. Please note that the HFD domain binding reduces the energy required for stripping the actin/cofilin complex from the pointed end by 86-107 ± 18 kJ/mol. (**e**) The frequency of contact (3 Å cutoff) between individual residues of each pair of actin molecule are shown for the HFD domains bound (left) and HFD-free filaments (right). The frequency of contacts was averaged over three simulations in each case. In the presence of the HFD domains, there were major reductions in the contact frequency between the pointed end actins A and B (upper left panel). The locations of these reductions are indicated by arrows on the upper left panel. However, the interfaces between non-pointed end actins (actin B – actin C, and actin C – actin D) in the presence and the absence of HFD were similar (panels on the rows 2 and 3). (**f**) The frequency of contact (3 Å cutoff) between individual residues of HFD and the pointed end actin molecules are shown. Each left panel shows the contact frequency of HFD domain residues with the corresponding actin molecule on the right panel. The residues involved in the mutagenesis experiments are indicated with arrows. The actin A residue that shows the most substantial contact frequency with HFD domain B is also labelled (lower right panel).

Supplementary Fig. 6

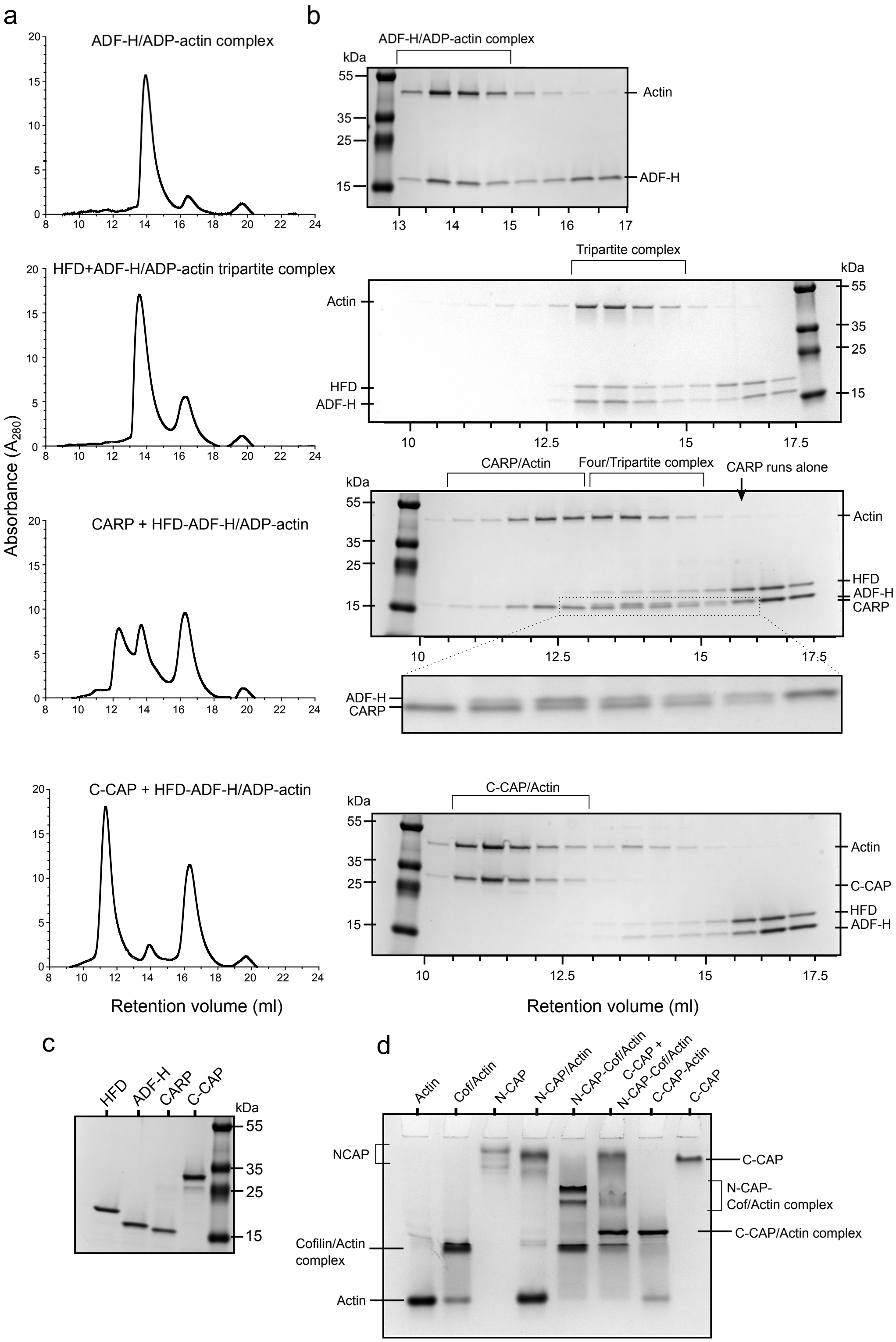

Supplementary Figure 6. Competition of N- and C-terminal halves of CAP for ADP-actin monomer binding. (**a**) Elution profiles of various protein combinations (of the HFD domain, C-terminal ADF-H domain of twinfilin, CARP domain, C-CAP, and ADP-actin) on a Superdex 200 increase 10/300 GL gel filtration column. Samples contained 18 µM ADF-H domain, and 15 µM of other proteins. 100 µl of sample was injected to and analyzed for retention. Fractions were collected at 0.5 mL intervals. (**b**) Peak fractions (from panel a) analyzed by SDS-PAGE. Please note that the ADF-H domain makes a complex with ADP-G-actin (top) and the HFD domain can from a tripartite complex with ADF-H domain and ADP-G-actin (second one form the top). The C-CAP (bottom) and to lesser extent the CARP domain (second one from the bottom) can dissociate this complex, and release ADF-H domain and N-CAP. **(c)** Migration of the proteins used in these experiments on SDS PAGE. Please note that the ADF-H domain of twinfilin and the isolated CARP domain of CAP migrate very close to each other on SDS-PAGE. (**d**) Native-PAGE analysis of the competition between N-CAP and C-CAP for binding to ADP-G-actin. N-CAP can form a tripartite complex with cofilin/ADP-G-actin. Addition of C-CAP dissociates N-CAP form actin and leads to the formation of C-CAP/ADP-G-actin complex. Please note that cofilin alone does not enter to the gel due to its high isoelectric point. Samples contained 10 µM each proteins, of which of 5 µl were loaded on the gel.

Supplementary Fig. 7

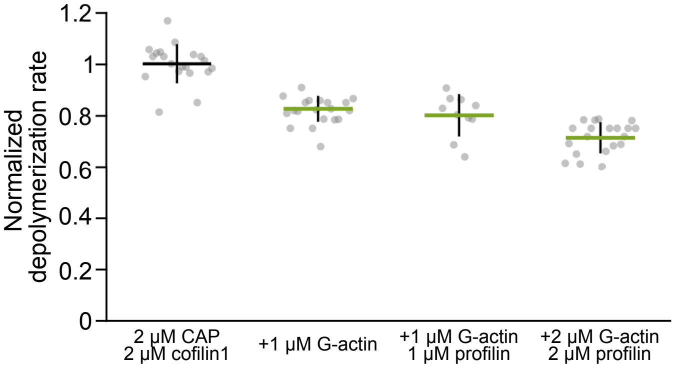

Supplementary Figure 7. Full-length CAP accelerates pointed depolymerization in the presence of ATP-G-actin and profilin. The pointed end depolymerization of cofilin-1 saturated filaments was measured as in Fig. 2, and normalized to the one in the absence of G-actin and profilin. n = 20 filaments were analyzed for each condition except "+ 1 µM G-actin, 1 µM profilin", where 10 filaments were analyzed. Horizontal lines represent mean values, and vertical lines S.D.

SUPPLEMENTARY TABLES

Supplementary Table 1. The initial box vectors, the number of water molecules, and ions, and the length of the simulations

Systems	No.	Initial Box Vector / Å			N_{water}	N_{Na}	N_{Cl}	Length / μs	Initial Configuration
		X	Y	Z					
HFDs bound	C1	147	123	223	100000	306	233	2.03	model 1
	C2	146	123	223	100000	306	233	2.01	model 2
	C3	146	123	223	100000	306	233	1.61	model 3
	C1'	145	125	220	100000	306	233	1.97	F1 at t = 2.42 μs
	C2'	145	125	221	100000	306	233	2.32	F2 at t = 0.94 μs
HFD-free	F1	145	126	196	90000	279	206	2.42	model 1
	F2	146	126	196	90000	279	206	2.28	model 2
	F3	146	125	197	90000	279	206	1.12	model 3
	F2'	146	123	201	90000	279	206	2.11	C2 at t = 0.76 μs

The last column specifies the source of the initial configuration. Models 1-3 are the top scoring cofilin-decorated actin filaments constructed as described in Methods. The HFD bound systems, C1' and C2', were generated by docking HFD and the HFD-free system, F2', was generated by removing HFD starting from the indicated configurations.

Supplementary Table 2. The DNA constructs, primers and methods for cloning in this study. Mutations and linking sequences are italicized.

Plasmid	Protein	Primers used for cloning	Vector	Template	Method
pPL973	PP1+WH2+PP2+CARP domains of mouse CAP1 (*GP* + 217-474)	3C_mmCAP_1_F, ccdB_mmCAP_1_R, ccdB_F, 3C_R	pCoofy18	pPL216[6]	[7]
pPL975	CARP domain of mouse CAP1 (*GP* + 318-474)	3C_mmCAP_3_F, ccdB_mmCAP_1_R, ccdB_F, 3C_R	pCoofy18	pPL216	[7]
pPL1063	HFD domain (42-216) of mouse CAP1	mHFDF2, mHFDR2, pPL989_del_fwd, pPL989_del_rev	pCoofy18	pPL989, pPL216	[7], mutagenesis
pPL1337	HFD domain (*C92S*, 39-210) of mouse CAP1	CAP_2_F, CAP_210_R, CAP_C92S_F, CAP_C92S_R, pSUMO_F, pSUMO_R	pSUMOck3	pPL216	NEBuilder, mutagenesis
pPL1340	C-terminal ADF-H domain of mouse twinfilin (176-316)	Twf_176_F, Twf_316_R, pSUMO_F, pSUMO_R	pSUMOck4	pPL846[8]	NEBuilder
pPL1363	N-CAP (2-210) of mouse CAP1	CAP_2_F, CAP_210_R, pSUMO_F, pSUMO_R	pSUMOck4	pPL216	NEBuilder
pPL1364	HFD domain (39-210) of mouse CAP1	CAP_39_F, CAP_210_R, pSUMO_F, pSUMO_R	pSUMOck4	pPL216	NEBuilder
pPL1665	N-CAP (2-210) *F162A+Y163A*	NCAPm1_f, NCAPm1_r	pPL1363	pPL1363	Mutagenesis
pPL1666	N-CAP (2-210) *K154A+E155A+D158S*	NCAPm2_f, NCAPm2_r	pPL1363	pPL1363	Mutagenesis
pPL1667	N-CAP (2-210) *K197D+T202D*	NCAPm3_f,NCAPm3_r	pPL1363	pPL1363	Mutagenesis
pPL1358	Full length mouse CAP1 (2-474)	CAP_2_F, CAP_474_R, pSUMO_F, pSUMO_R	pSUMOck4	pPL216	NEBuilder
pPL1459	N-CAP (2-210)-*SSGSSG*-sfGFP	NCAP-GFP_f, NCAP-GFP_r, sfGFP_f, sfGFP_r	pSUMOck4	pPL1363, pPL1155	NEBuilder

Primer	Sequence 5'->3'	Primer	Sequence 5'->3'
**CAP_2_F**	AGCAGCAGACGGGAGGGGCTGACATGCAAAATCTTGTAGAAAGATTGGAGAG	**Twf_176_F**	AGCAGCAGACGGGAGGGCAAGGCGTAGCCTTTCCTATTTCTCGAGATG
**CAP_39_F**	AGCAGCAGACGGGAGGGGCAGTTCCATATGTGCAAGCATTTGACTCG	**Twf_316_R**	CTTTGTTAGCAGCCGGATCTCACTGCTTGGGGTGGACTTCATCGTACAG
**CAP_210_R**	CTTTGTTAGCAGCCGGATCTCACCCCGTCTTGCTCCAGGCCA	**mHFDF2**	GCAAAAGAACTGTAAGCACTCGAGCACCACCACCACCACCACTG
**CAP_474_R**	CTTTGTTAGCAGCCGGATCTCATCCAGCGATTTCTGTCACTGTGGTG	**mHFDR2**	GCTCGAGTGCTTACAGTTCTTTTGCCACAGGCCCCGTCTTGC
**CAP_C92S_F**	CAGCTTCTCAGAGCCAGCAGCCAGCTGGTAATAAAC	**pPL989_del_fwd**	CCTGTGGCAAAAGAACTGTAAGCACTCGAGCACCACCAC
**CAP_C92S_R**	TGCTGGCTCTGAGAAGCTGTAGCCAGGAGAGC	**pPL989_del_rev**	CAGTTCTTTTGCCACAGGCCCCGTCTTGCTCCAGGC
**pSUMO_F**	TGAGATCCGGCTGCTAACAAAGCCC	**NCAPm1_f**	CCATGGCTGCCACAAATCGTGTCCTCAAGG
**pSUMO_R**	CCCTCCCGTCTGCTGCTGGA	**NCAPm1_r**	GATTTGTGGCAGCCATGGCCGCGTC
**3C_mmCAP_1_F**	CTGGAAGTTCTGTTCCAGGGGCCCAGTGGATTGCCATCTGGACCCTC	**NCAPm2_f**	GGCAGCGATGAATAGCGCGGCCATGTTTTAC
**3C_mmCAP_3_F**	CTGGAAGTTCTGTTCCAGGGGCCCCCAGCTCTGCTGGAACTGGAAG	**NCAPm2_r**	CGCTATTCATCGCTGCCACAAAGGGGCCAG
**ccdB_mmCAP_1_R**	CCCCAGAACATCAGGTTAATGGCGCTATCCAGCGATTTCTGTCACTGTGG	**NCAPm3_f**	CGATGAGTTTCATACTGATGGCCTGGCC
**ccdB_F**	CGCCATTAACCTGATGTTCTGGGG	**NCAPm3_r**	CATCAGTATGAAACTCATCGATGTAAGCCTGCAGC
**3C_R**	GGGCCCCTGGAACAGAACTTCCAG		
**NCAP-GFP_f**	CATGGACGAGCTGTACAAGTGAGATCCGGCTGCTAACAAAGCCC	**sfGFP_f**	AGTAGCGGTTCAAGCGGCGTGAGCAAGGGCGAGGAGCTGTTC
**NCAP-GFP_r**	GCCGCTTGAACCGCTACTCCCCGTCTTGCTCCAGGCCAG	**sfGFP_r**	CTTGTACAGCTCGTCCATGCCGAGAGTG

SUPPLEMENTARY REFERENCES

1. Quintero-Monzon, O. *et al.* Reconstitution and dissection of the 600-kDa Srv2/CAP complex: Roles for oligomerization and cofilin-actin binding in driving actin turnover. *J. Biol. Chem.* **284**, 10923–10934 (2009).
2. Normoyle, K. P. M. & Brieher, W. M. Cyclase-associated Protein (CAP) Acts Directly on F-actin to Accelerate Cofilin-mediated Actin Severing across the Range of Physiological pH. *J. Biol. Chem.* **287**, 35722–35732 (2012).
3. Johnston, A. B., Collins, A. & Goode, B. L. High-speed depolymerization at actin filament ends jointly catalysed by Twinfilin and Srv2/CAP. *Nat. Cell Biol.* **17**, 1504–1511 (2015).
4. Chaudhry, F. *et al.* Srv2/cyclase-associated protein forms hexameric shurikens that directly catalyze actin filament severing by cofilin. *Mol. Biol. Cell* **24**, 31–41 (2013).
5. Kotila, T. *et al.* Structural basis of actin monomer re-charging by cyclase-associated protein. *Nat. Commun.* **9**, 1892 (2018).
6. Bertling, E. *et al.* Cyclase-associated Protein 1 (CAP1) Promotes Cofilin-induced Actin Dynamics in Mammalian Nonmuscle Cells. *Mol. Biol. Cell* **15**, 2324–2334 (2004).
7. Scholz, J., Besir, H., Strasser, C. & Suppmann, S. A new method to customize protein expression vectors for fast, efficient and background free parallel cloning. *BMC Biotechnol.* **13**, 12 (2013).
8. Paavilainen, V. O., Oksanen, E., Goldman, A. & Lappalainen, P. Structure of the actin-depolymerizing factor homology domain in complex with actin. *J. Cell Biol.* **182**, 51–59 (2008).
