# Supplementary material for: Mechanism of synergistic actin filament pointed end depolymerization by cyclase-associated protein and cofilin
Source: Nat Commun. 2019 Nov 22;10:5320. doi: 10.1038/s41467-019-13213-2 (PMC6876575; doi:10.1038/s41467-019-13213-2)
Supplement: Supplementary file 3 — Reporting Summary [file 41467_2019_13213_MOESM3_ESM.pdf]

nature research

Corresponding author(s): Pekka Lappalainen

Last updated by author(s): Oct 11, 2019

Reporting Summary

Nature Research wishes to improve the reproducibility of the work that we publish. This form provides structure for consistency and transparency in reporting. For further information on Nature Research policies, see Authors & Referees and the Editorial Policy Checklist .

Statistics

For all statistical analyses, confirm that the following items are present in the figure legend, table legend, main text, or Methods section.

n/a	Confirmed	
☐	☒	The exact sample size (*n*) for each experimental group/condition, given as a discrete number and unit of measurement
☐	☒	A statement on whether measurements were taken from distinct samples or whether the same sample was measured repeatedly
☐	☒	The statistical test(s) used AND whether they are one- or two-sided<br>*Only common tests should be described solely by name; describe more complex techniques in the Methods section.*
☒	☐	A description of all covariates tested
☒	☐	A description of any assumptions or corrections, such as tests of normality and adjustment for multiple comparisons
☒	☐	A full description of the statistical parameters including central tendency (e.g. means) or other basic estimates (e.g. regression coefficient) AND variation (e.g. standard deviation) or associated estimates of uncertainty (e.g. confidence intervals)
☒	☐	For null hypothesis testing, the test statistic (e.g. *F*, *t*, *r*) with confidence intervals, effect sizes, degrees of freedom and *P* value noted<br>*Give P values as exact values whenever suitable.*
☒	☐	For Bayesian analysis, information on the choice of priors and Markov chain Monte Carlo settings
☒	☐	For hierarchical and complex designs, identification of the appropriate level for tests and full reporting of outcomes
☒	☐	Estimates of effect sizes (e.g. Cohen's *d*, Pearson's *r*), indicating how they were calculated

Our web collection on statistics for biologists contains articles on many of the points above.

Software and code

Policy information about availability of computer code

Data collection	All information concerning the versions and softwares to determine the crystal structures are listed in Protein Data Bank under the reported accession codes. All scripts used for analysis of the simulations will be available in Zenodo along with all the raw data.
Data analysis	Data were analysed and plotted with Microsoft Excel, Prism 8 and ImageJ 1.52a.

For manuscripts utilizing custom algorithms or software that are central to the research but not yet described in published literature, software must be made available to editors/reviewers. We strongly encourage code deposition in a community repository (e.g. GitHub). See the Nature Research guidelines for submitting code & software for further information.

Data

Policy information about availability of data

All manuscripts must include a data availability statement. This statement should provide the following information, where applicable:

- Accession codes, unique identifiers, or web links for publicly available datasets
- A list of figures that have associated raw data
- A description of any restrictions on data availability

The crystallographic data are stored in Protein Data Bank under accession codes 6RSQ and 6RSW. The molecular dynamics simulation data are available from Zenodo (doi: 10.5281/zenodo.3340994). All other data are available from the corresponding author upon reasonable request.

Field-specific reporting

Please select the one below that is the best fit for your research. If you are not sure, read the appropriate sections before making your selection.

Life sciences study design

All studies must disclose on these points even when the disclosure is negative.

Sample size	We did not use any statistical tests to predetermine sample size.
Data exclusions	In the analysis of rates of single actin filaments (depolymerization from pointed ends), we excluded filaments whose pointed ends could not be clearly tracked or which exhibited severing events or pauses.
Replication	There were no experiments, which could not be replicated.
Randomization	N/A
Blinding	In the experiments measuring the binding of GFP-N-CAP to the filament ends, we first blindly selected actin filaments that did not move. The blindly selected filaments were analyzed for binding of N-CAP by visualizing the GFP-channel.

Reporting for specific materials, systems and methods

We require information from authors about some types of materials, experimental systems and methods used in many studies. Here, indicate whether each material, system or method listed is relevant to your study. If you are not sure if a list item applies to your research, read the appropriate section before selecting a response.

Materials & experimental systems

n/a	Involved in the study
☒	☐ Antibodies
☒	☐ Eukaryotic cell lines
☒	☐ Palaeontology
☒	☐ Animals and other organisms
☒	☐ Human research participants
☒	☐ Clinical data

Methods

n/a	Involved in the study
☒	☐ ChIP-seq
☒	☐ Flow cytometry
☒	☐ MRI-based neuroimaging
