# Supplementary material for: Mechanism of synergistic actin filament pointed end depolymerization by cyclase-associated protein and cofilin
Source: Nat Commun. 2019 Nov 22;10:5320. doi: 10.1038/s41467-019-13213-2 (PMC6876575; doi:10.1038/s41467-019-13213-2)
Supplement: Supplementary file 4 — Description of Additional Supplementary Files [file 41467_2019_13213_MOESM4_ESM.pdf]

Title: Supplementary Movie 1.

Description: Molecular dynamics simulations of actin filament pointed end in the presence and absence of HFD domains. The motion of the ultimate actin relative to the penultimate actin at the filament pointed end. Representative simulation trajectories for the HFD domain bound actin filament (left) and the HFD-free actin filament (right) are presented. The ultimate (blue) and the penultimate (green) actin molecules, as well as the HFD domains (magenta) are depicted in ribbon representation. All other components of the simulation systems were omitted for clarity, and the systems are shown from the top view. The separation between the subdomain 2 of the penultimate actin and the subdomain 4 of the ultimate actin increases in the HFD domain bound simulations resulting in decreased number of contacts between them. In contrast, the interface between the two actins remains stable throughout the simulations in the absence of bound HFD domains.
