# Supplementary material for: Mechanism of synergistic actin filament pointed end depolymerization by cyclase-associated protein and cofilin
Source: Nat Commun. 2019 Nov 22;10:5320. doi: 10.1038/s41467-019-13213-2 (PMC6876575; doi:10.1038/s41467-019-13213-2)
Supplement: Supplementary file 5 — Peer Review File [file 41467_2019_13213_MOESM5_ESM.pdf]

Reviewers' comments:

Reviewer #1 (Remarks to the Author):

Using biochemical reconstitution assays in combination with structural methods, Kotilla et al. establish a detailed mechanism of actin depolymerization meditated by the synergy between two conserved proteins -CAP and cofilin- previously implicated in actin disassembly. Overall, this is one of the most interesting manuscripts I have read in a while and the experiments are of high quality throughout. I enthusiastically support publication of the paper. My recommendations below are for the most part minor, and are mostly intended to enrich the manuscript.

1. My most important concern relates to the integration and coordination of the versatile activities of CAP in the context of the full length protein. Most of the work is done with an N-terminal fragment of CAP in the absence of bare actin monomers or profilin-actin complexes in solution. Soluble actin species are, however, present at considerable concentrations in vivo and CAP can interact with at least one of these through its C-terminal domain. It would be interesting to learn whether depolymerization mediated by full length CAP is affected/inhibited by the presence of monomeric actin and/or profilin-actin complexes in solution.

2. The finding that N-CAP does not stimulate cofilin-mediated severing (Suppl Fig. 3C-D) is an important one and deserves to be moved to the main part of the manuscript in my opinion. Is the same true for the full length molecule? Previous studies have claimed that CAP exerts its role by enhancing severing by cofilin (Chaudhry et al 2013). It would be good if the authors could comment on and more directly discuss the differences/similarities between their own and past work by others.

3. Oligomerization of CAP seems to be required for the high affinity interaction with cofilin-decorated pointed ends, but not for acceleration of depolymerization per se. I therefore find it misleading to state that the isolated HFD is "less efficient". I believe that is important to point out that the HFD is the functionally autonomous unit for depolymerization that can provide similar rapid rates at saturation.

4. It would be interesting to learn whether individual CAP molecules remain bound to the pointed end they depolymerize in a processive manner. If they do, how do CAP oligomerization state and the presence of soluble actin species affect processivity?

Reviewer #2 (Remarks to the Author):

This manuscript discovers the actin-pointed end depolymerization role of cyclase-associated protein (CAP). The authors determine the crystal structures of the HDF domain alone and in complex with actin and cofilin. These structures provide the basis for the hypothesis that CAP/cofilin depolymerize actin filaments from the pointed. Biochemical assays substantiate this hypothesis, with some particularly compelling data provided by TIRF microscopy. Together, these data represent a substantial advance in our understanding of cellular actin dynamics. I recommend publication after the following minor issues are rectified.

Major point:
The model presented in Figure 7 is highly testable in the TIRF assay. CAP an be anchored to the microchamber surface in the TIRF assay. Gelsolin capped filaments can be captured by the CAP and the depolymerization observed. This will be particularly convincing if two-color filaments are used. ie polymerize red actin from gelsolin, then polymerize green actin from the red actin/gelsolin seeds.

Minor issues:
1) The English could be improved – for instance line 7 page 4 "actin pointed end"

p5 L24 "only quite"
p5 L22 "un-optimal"
p6 L30 "through avidity effect"
p7 L11 "Largest"
p3 L1 "outstanding open" only one term needed

2) Abstract – "innovation" is a poor choice of word "adaptation" is better
3) Abstract – Ubiquitous should be qualified "in eukaryotes"
4) Introduction – There no mention of the gelsolin family, which skews the discussion of actin recycling in cells. This is particularly surprising since it is used as a reagent in the TIRF assay.
5) P4/L4 "identical" is not correct
6) P4/L22 "bare actin" is not correct. 1J6Z is modified.
7) P5/L2 – reference 36 is an EM structure of the central portion of a cofilin-decorated filament. It does not show the pointed-end structure. Similarly, P5/L4 does not show the pointed end of a bare actin filament. The authors need to state their assumption that the ends have the same conformation to the central portions in the EM structures. There is some evidence that this is not the case: EMBO J. 2011 Apr 6;30(7):1230
8) There was a long running controversy in the Arp2/3 field whether Arp2/3 binding happens at the end or close to the barbed end of actin filament. Similarly, can the authors rule out, and comment upon, whether CAP can remove multiple actin-subunits in each step? Ie could it be severing close to the pointed end?
9) Can the authors comment on the cellular concentration of CAP relative to filament pointed ends?
10) The discrepancy with previous reports of CAP severing should be discussed. Refs 28 and 32

Reviewers' comments:

Reviewer #1 (Remarks to the Author):

Using biochemical reconstitution assays in combination with structural methods, Kotilla et al. establish a detailed mechanism of actin depolymerization meditated by the synergy between two conserved proteins -CAP and cofilin- previously implicated in actin disassembly. Overall, this is one of the most interesting manuscripts I have read in a while and the experiments are of high quality throughout. I enthusiastically support publication of the paper. My recommendations below are for the most part minor, and are mostly intended to enrich the manuscript.

We thank the reviewer for positive feedback on our manuscript.

1. My most important concern relates to the integration and coordination of the versatile activities of CAP in the context of the full length protein. Most of the work is done with an N-terminal fragment of CAP in the absence of bare actin monomers or profilin-actin complexes in solution. Soluble actin species are, however, present at considerable concentrations in vivo and CAP can interact with at least one of these through its C-terminal domain. It would be interesting to learn whether depolymerization mediated by full length CAP is affected/inhibited by the presence of monomeric actin and/or profilin-actin complexes in solution.

This is an interesting point. We performed the suggested experiment with a new batch of full-length CAP in the presence and absence of G-actin/profilin complexes. We observed only quite modest effects on the pointed end depolymerisation (when using up to 2 uM G-actin/profilin), suggesting that CAP can efficiently depolymerize cofilin-decorated actin filaments also in the presence of soluble profilin/actin species. The new data are presented in Supplementary Fig. 7, and discussed on page 10.

2. The finding that N-CAP does not stimulate cofilin-mediated severing (Suppl Fig. 3C-D) is an important one and deserves to be moved to the main part of the manuscript in my opinion.

Our experiments provide evidence that the N-terminal half of mouse CAP1 does not enhance severing of partially cofilin-decorated actin filaments. However, these data do not rule out the possibility that other members of this protein family could affect actin filament severing. For this reason, and because these findings partially diverge from the main point of our manuscript (actin filament pointed end depolymerisation by CAP), we prefer to present these data in the supplementary information. If necessary, we can also move the severing data to the main part of the manuscript.

Is the same true for the full length molecule?

We performed additional experiments to measure the severing rate at cofilin domain boundaries, in the presence of full-length CAP1. In these experiments, we detected only a very modest (<50 %) increase in the severing rate, suggesting that the full-length CAP does not induce robust actin filament severing either, in contrast to earlier suggestions. Please, note that in previous studies CAP was reported to accelerate cofilin-dependent severing by up to 8-fold (e.g. Jansen et al., 2014). The small increase in the filament severing observed in the presence of full-length CAP in our experiments may arise e.g. from 'unspecific' anchoring of actin filaments to the coverslip by CAP (full-length CAP appears to stick more to the coverslip than its N-terminal fragment), which would constrain their twist and hence induce filament severing as described in Wioland *et al.*, (PNAS, 2019). These new data are shown in the new version of Supplementary Figure 3 (panel c), and discussed in the manuscript text on page 10.

Previous studies have claimed that CAP exerts its role by enhancing severing by cofilin (Chaudhry et al 2013). It would be good if the authors could comment on and more directly discuss the differences/similarities between their own and past work by others.

The main difference between our experiments and previous reports is that we do not anchor filaments along their length to the coverslip surface. We think this might have caused two issues in interpreting the data in previous reports:

1) Severing events can be more difficult to detect when filaments are anchored, as fragments are restricted from diffusing away. It might be that accelerated pointed end depolymerization by CAP helped to reveal the severing events and thus might explain the larger severing rates previously reported.
2) We have recently shown (Wioland *et al.* 2019) that severing is highly enhanced when filaments are anchored. This might further lead to over-interpretations in the above mentioned case.

We have now discussed these points in our manuscript at p. 10.

Nevertheless, our work, together with a related manuscript by Bruce Goode's lab (which is also submitted to Nature Communications), provide strong evidence that the main function of CAP in actin filament turnover is to promote filament pointed end depolymerisation, and subsequently 're-charge' actin monomers by nucleotide exchange.

3. Oligomerization of CAP seems to be required for the high affinity interaction with cofilin-decorated pointed ends, but not for acceleration of depolymerization per se. I therefore find it misleading to state that the isolated HFD is "less efficient". I believe that is important to point out that the HFD is the functionally autonomous unit for depolymerization that can provide similar rapid rates at saturation.

This is a good point. We have accordingly rephrased our manuscript (page 7) to state that the isolated HFD domain appears to bind pointed ends with lower affinity, but can nevertheless reach similar level of depolymerisation activity as oligomeric N-CAP.

4. It would be interesting to learn whether individual CAP molecules remain bound to the pointed end they depolymerize in a processive manner. If they do, how do CAP oligomerization state and the presence of soluble actin species affect processivity?

This is an interesting point. We accordingly tried to perform such experiments with GFP-CAP (full-length) and GFP-N-CAP. We observed GFP-N-CAP dissociating from the pointed ends of cofilin-1 –decorated actin filaments with a k_{off} rate of 1.7 s^{-1}, but this corresponds to a residence time that is too short for a measurable depolymerization to take place (please see the data in Fig. 2g and h of the revised manuscript). The same experiments with GFP-CAP were technically impossible as the fusion protein had a tendency to aggregate and stick to the coverslips. Thus it was not possible to reliably follow its association with filament pointed ends, and get reliable data on the processivity. This is now discussed on pages 9-10 of the manuscript text. Please note that a related/complementary manuscript by Bruce Goode lab (also submitted to Nat. Comm.) describes a more extensive analysis of processivity of CAP at filament pointed end.

Reviewer #2 (Remarks to the Author):

This manuscript discovers the actin-pointed end depolymerization role of cyclase-associated protein (CAP). The authors determine the crystal structures of the HDF domain alone and in complex with actin and cofilin. These structures provide the basis for the hypothesis that CAP/cofilin depolymerize actin filaments from the pointed. Biochemical assays substantiate this hypothesis, with some particularly compelling data provided by TIRF microscopy. Together, these data represent a substantial advance in our understanding of cellular actin dynamics. I recommend publication after the following minor issues are rectified.

We thank the reviewer for the positive comments on our study.

Major point:
The model presented in Figure 7 is highly testable in the TIRF assay. CAP an be anchored to the microchamber surface in the TIRF assay. Gelsolin capped filaments can be captured by the CAP and the depolymerization observed. This will be particularly convincing if two-color filaments are used. ie polymerize red actin from gelsolin, then polymerize green actin from the red actin/gelsolin seeds.

This is an interesting suggestion, and we accordingly tried to perform such experiments to examine the processivity of CAP-driven actin filament depolymerisation. For such experiments, we would need to immobilize fluorescent full-length GFP-CAP on coverslips (in order to detect if the actin filaments indeed associate with CAP when they are captured on the coverslip). Our experiments revealed that full-length GFP-CAP has a tendency to aggregate, thus making it difficult to reliably follow the depolymerisation of those filaments that have CAP bound to their pointed end. As an alternative approach to study the duration of CAP-binding to depolymerizing actin filament pointed ends, we imaged GFP-fusions of full-length CAP and N-CAP together with actin filaments. As described in response to point #4 by reviewer #1, we observed transient GFP-N-CAP binding to filament pointed end (k_{off} ~1.7 s^{-1}; see also manuscript text pages 5 and 6, as well as Fig. 2g and h). Because the full-length GFP-CAP had a tendency to aggregate and 'unspecifically' bind to the coverslip, we could not reliably measure its residence time at filament pointed ends (or follow pointed end depolymerisation of actin filaments in which CAP is bound). We now explain in the 'Discussion' (pages 9-10) that future work is needed to determine the residence time of full-length CAP at the filament pointed end, and how processive filament depolymerase this protein is. Moreover, we are aware that a related/complementary manuscript by Bruce Goode lab has been submitted to Nature Communications and it describes a more extensive analysis of processivity of CAP at filament pointed end.

Minor issues:
1) The English could be improved – for instance line 7 page 4 "actin pointed end"
p5 L24 "only quite"
p5 L22 "un-optimal"
p6 L30 "through avidity effect"
p7 L11 "Largest"
p3 L1 "outstanding open" only one term needed

We have corrected these in the manuscript text.

2) Abstract – "innovation" is a poor choice of word "adaptation" is better

We agree and have accordingly modified this in the text.

3) Abstract – Ubiquitous should be qualified "in eukaryotes"

We have corrected this.

4) Introduction – There no mention of the gelsolin family, which skews the discussion of actin recycling in cells. This is particularly surprising since it is used as a reagent in the TIRF assay.

We apologize for ignoring the gelsolin family of proteins. We have now briefly introduced gelsolin family proteins in the 'Introduction', page 2.

5) P4/L4 "identical" is not correct

We have rephrased this to "similar".

6) P4/L22 "bare actin" is not correct. 1J6Z is modified.

This has now been corrected in the text.

7) P5/L2 – reference 36 is an EM structure of the central portion of a cofilin-decorated filament. It does not show the pointed-end structure. Similarly, P5/L4 does not show the pointed end of a bare actin filament. The authors need to state their assumption that the ends have the same conformation to the central portions in the EM structures. There is some evidence that this is not the case: EMBO J. 2011 Apr 6;30(7):1230

We agree with these points, and have accordingly modified the manuscript text (page 6).

8) There was a long running controversy in the Arp2/3 field whether Arp2/3 binding happens at the end or close to the barbed end of actin filament. Similarly, can the authors rule out, and comment upon, whether CAP can remove multiple actin-subunits in each step? Ie could it be severing close to the pointed end?

This is an interesting point. From our experiments it is not possible to directly interpret whether individual or multiple actin subunits are removed in each cycle, but we have several arguments supporting that only one subunit is removed at each cycle:

1) In MD simulations, we only observed the 'weakening' of the interface between the two terminal actin subunits in the presence of HFD domains, while the interfaces between other actin subunits we not significantly affected (see e.g. Supplementary Fig. 5e). This is now also discussed on page 8.
2) We think that it is unlikely that CAP would 'sever' filament close to the pointed end, because based on our structural data the HFD domain cannot bind to the side of bare or cofilin-decorated actin filament.
3) Finally, a known artefact in the experimental setup we use to image depolymerization of actin filaments is the formation of photo-induced actin dimers that cause pauses in the filament depolymerization (Niedermayer *et al.* 2012). We have observed that N-CAP cannot depolymerize these filaments, which undergo a pause. However, when the crosslinked dimer finally dissociates, depolymerization continues (see enclosed figure below). Also this experiment indicates that N-CAP does not enhance depolymerization e.g by binding to the side of the filament and causing severing close to the pointed end.

9) Can the authors comment on the cellular concentration of CAP relative to filament pointed ends?

Both Johnston *et al.* (2015) and Bertling *et al.* (2004) studies provided evidence that CAP is an abundant protein, and exists in ~1:4 ratio to actin in cells. Raz-Ben Aroush *et al.* (2017) study suggests that the concentration of pointed ends in cells is in low micromolar range, whereas the amount of total actin is in millimolar range. Thus, from these data CAP should be in large excess to actin filament pointed ends in the cytoplasm. This is now discussed on page 10.

10) The discrepancy with previous reports of CAP severing should be discussed. Refs 28 and 32

We have now extended discussion on the previous reports of CAP as a protein enhancing actin filament severing (see page 10).

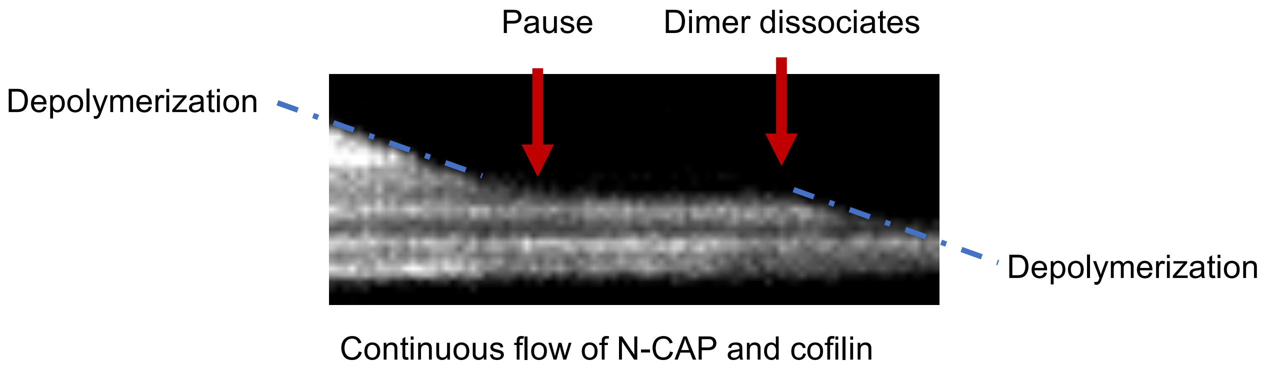

Enclosed figure. A kymograph of cofilin-decorated actin filament pointed end depolymerisation, which demonstrates that N-CAP cannot depolymerize actin filaments which undergo a pause due to formation of stable photoinduced dimers (Niedermayer *et al.* 2012). When the dimer dissociates, N-CAP is able to continue depolymerization of a cofilin-decorated actin filament.

REVIEWERS' COMMENTS:

Reviewer #1 (Remarks to the Author):

The authors have comprehensively addressed the points raised in the initial round of review. I fully support its publication.

Reviewer #2 (Remarks to the Author):

The authors have made every effort to address this referee's comments. This manuscript is a major contribution to the understanding of CAP function. I recommend publication without further change.
